# A randomized, phase II trial of oral azacitidine (CC-486) in patients with resected pancreatic adenocarcinoma at high risk for recurrence

**DOI:** 10.1186/s13148-022-01367-8

**Published:** 2022-12-03

**Authors:** Thatcher R. Heumann, Marina Baretti, Elizabeth A. Sugar, Jennifer N. Durham, Sheila Linden, Tamara Y. Lopez-Vidal, James Leatherman, Leslie Cope, Anup Sharma, Colin D. Weekes, Peter J. O’Dwyer, Kim A. Reiss, Dulabh K. Monga, Nita Ahuja, Nilofer S. Azad

**Affiliations:** 1 The Sidney Kimmel Comprehensive Cancer Center at Johns Hopkins, Baltimore, MD USA; 2 Departments of Biostatistics and Epidemiology, The Bloomberg School of Public Health at Johns Hopkins, Baltimore, MD USA; 3 Yale Cancer Center, Yale University School of Medicine, New Haven, CT USA; 4 Massachusetts General Hospital Cancer Center, Boston, MA USA; 5 Abramson Cancer Center, University of Pennsylvania, Philadelphia, PA USA; 6 Medical Oncology, Allegheny General Hospital, Pittsburgh, PA USA

**Keywords:** Pancreatic cancer, Epigenetic therapy, Clinical trial, Hypomethylation, Azacitidine, Maintenance therapy

## Abstract

**Background:**

Of the only 20% of patients with resectable pancreatic ductal adenocarcinoma (rPDA), cancer recurs in 80% of cases. Epigenetic dysregulation is an early hallmark of cancer cells acquiring metastatic potential, and epigenetic modulators may reactivate tumor suppressor genes, delay recurrence, and sensitize PDA to future chemotherapy.

**Methods:**

This was a randomized phase II study (NCT01845805) of CC-486 (oral DNA methyltransferase inhibitor azacitidine) vs. observation (OBS) in rPDA patients harboring high-risk features (stage pN1-2, R1 margins, or elevated CA 19–9 level) with no evidence of disease following standard adjuvant therapy. Patients were randomized to oral CC-486 treatment (300 mg daily on days 1–21 on a 28-day cycle) or OBS for up to 12 cycles or until disease relapse/unacceptable toxicities. Following recurrence, records of next-line therapies, imaging, and survival were obtained. The primary endpoint was progression-free survival (PFS)—time from randomization to recurrence (imaging/biopsy confirmed or death). Secondary endpoints included OS and PFS and ORR and metastatic PFS with subsequent next-line systemic therapy in metastatic setting.

**Results:**

Forty-nine patients (24 in CC-486 arm, 25 in OBS arm) were randomized: median age 66 (range 36–81), 53% male, 73% node positive, 49% elevated CA 19–9, 20% R1 resection, 63% and 100% received perioperative concurrent chemoradiation and chemotherapy, respectively. Median time from surgery to randomization was 9.6 mo (range 2.9–36.8). For the CC-486 arm, median treatment duration was 5.6 mo (range 1.3 to 12.8) with 14 treatment-related grade 3 or 4 AEs among 5 patients (22%) resulting in dose-reduction. Four patients (17%) discontinued therapy due to AEs. With median follow-up of 20.3mo (IQR 12.8, 41.4), 38 (79%) of evaluable patients recurred (34 imaging-confirmed, 4 clinically). Median PFS in imagining-confirmed cases was 9.2 and 8.9mo (HR 0.94, 95% CI 0.46–1.87, *p* = 0.85) for CC-486 and OBS patients, respectively. Median OS (2-yr OS%) was 33.8 (50%) and 26.4 mo (61%) in CC-486 and OBS patients, respectively. (HR 0.98, 95% CI 0.46–2.05, *p* = 0.96). ORR with subsequent chemotherapy in the metastatic setting was minimal in both arms.

**Conclusions:**

Treatment with CC-486 following adjuvant therapy did not prolong time-to-relapse in patients with high-risk rPDA or improve disease response on 1st-line metastatic therapy.

**Supplementary Information:**

The online version contains supplementary material available at 10.1186/s13148-022-01367-8.

## Introduction

Pancreatic ductal adenocarcinoma (PDA) has the highest case-fatality rate of any solid tumor. It projected to surpass colorectal cancer as the 2nd leading cause of cancer-related mortality nationally in the next 5 years [1, 2]. Even for the 15–20% of patients eligible for curative resection at diagnosis, their 5-yr overall survival remains discouraging at 20%, with > 80% of cases recurring just two-year postsurgical intervention [3]. Patients that are node positive or margin positive at the time of resection have a greater than 90% chance of recurrence and death from disease [4–8].

Epigenetic changes, such as DNA hypermethylation leading to inactivation of tumor suppressor genes, are integral part of PDA’s development, progression, intratumoral heterogeneity, tumor microenvironment dynamics, immune escape, and chemoresistance [9–11]. Hypomethylating agents, such as 5-azacitidine, can reverse DNA hypermethylation, allowing for reactivation of tumor suppressor genes and ultimately leading to cancer cell death. Additionally, treatment with hypomethylating agents can initiate the reprogramming of cancer cells and activate e multiple pathways, including immunomodulator and interferon responses that may sensitize cancer cells to therapies, including immunotherapeutic and systemic chemotherapy modalities [12, 13]. These agents are currently the part of the standard for myelodysplastic syndromes [14] and acute myeloid leukemia [15]. The oral formulation of azacitidine, CC-486, is bioavailable and well tolerated and produces similar clinical responses as its subcutaneous formulation [16, 17]**.**

In both PDA cell lines and murine models, treatment with hypomethylating agents, such as azacitidine, inhibits growth and sensitizes tumors to chemotherapy [18, 19]. The goal of this trial was to evaluate whether CC-486 was well tolerated and effective as consolidation adjuvant therapy in patients with resected PDA (rPDA) at increased risk for recurrence due to nodal disease, microscopic margin positive (R1) resection, and/or elevated CA 19–9 following completion of standard of care treatment.

## Methods

### Study overview

This trial, NCT01845805, was a randomized, open-label, phase II study of CC-486 versus observation (no placebo) in patients with resected pancreatic adenocarcinoma who have finished adjuvant chemotherapy and/or chemoradiation (or were deemed unable to receive adjuvant therapy) with no evidence of visible disease on imaging and features associated with high risk for recurrence (see patients selection subjection). This trial was opened at the Sidney Kimmel Comprehensive Cancer Center at Johns Hopkins University, Abramson Cancer Center at University of Pennsylvania, Massachusetts General Hospital, and Allegheny General Hospital.

Patients with resected pancreatic adenocarcinoma who had concluded adjuvant therapy or were deemed unable to receive adjuvant therapy with an elevated CA 19–9 (defined as two levels > the institutional upper limit of normal (ULN) taken at least 2 weeks apart), node positive (pathology staged), or resection margin positive disease were eligible. Other key inclusion criteria included an Eastern Cooperative Oncology Group performance status of 0 or 1, and adequate organ function as defined by absolute neutrophil count ≥ 1500 cells/μL, hemoglobin > 9 g/dL, platelet count ≥ 75 000 cells/μL, total bilirubin ≤ 1.5 × upper limit of normal, and serum creatinine ≤ 2.0 mg/dL.

**Fig. 1:**
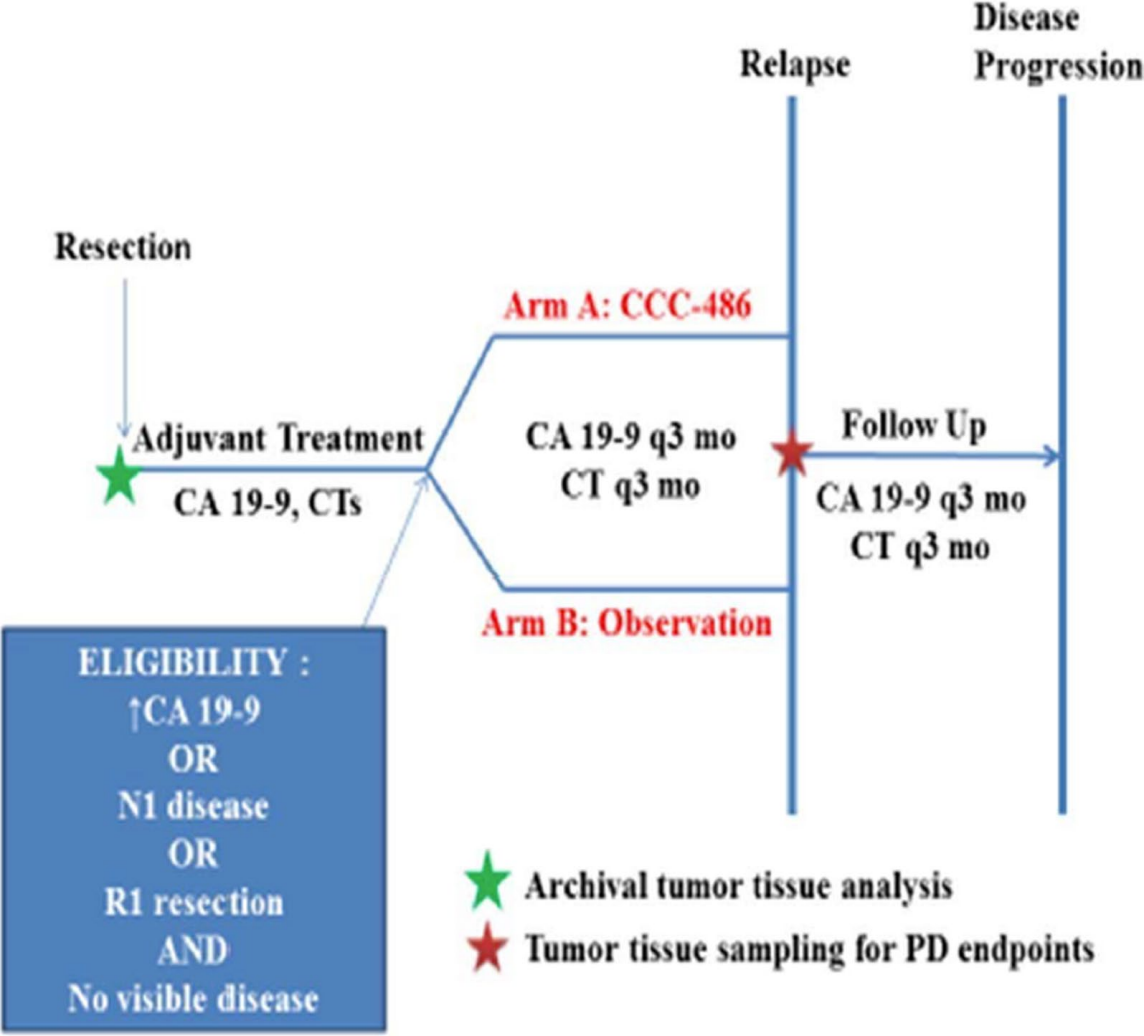
NCT01845805 Study Schema

Eligible and consented patients were randomized to one of two arms, stratified by node positive disease (Fig. 1): Arm A (received CC-486 at 300 mg daily by mouth for 21 days, repeated every 28 day cycle) and Arm B (observation only). Patients underwent CA 19–9 measurement and tumor assessment every three months. If their cancer recurred, Arm A participants stopped the CC-486, and Arm B participants stopped observation and began their respective next-line cancer treatment regimens as recommended by their primary managing oncologist. During this follow-up phase, we collected information regarding first-line advanced setting chemotherapy and survival outcomes (metastatic progression-free survival—MPFS and overall survival—OS) for all consenting patients from both arms.

### Clinical endpoints

The primary endpoint was progression-free survival (PFS). Secondary endpoints included response to subsequent first-line chemotherapy in the advanced disease setting, overall survival (OS), and toxicity. PFS was defined as the time from randomization until visible recurrence on any imaging modality, a confirmed biopsy, or death. Individuals lost to follow-up prior to having an event were censored at the time of the last tumor restaging scan. In the case of an initial equivocal scan reading with subsequent re-imaging confirming recurrence, the event was considered to have occurred at the first time point when a related abnormality was noted. Response to subsequent 1st-line chemo was assessed both as overall (best) response (partial [PR] or complete [CR]) rate (ORR) and metastatic PFS (MPFS) defined as time from start metastatic setting chemo to progression on that regimen or last follow/death. Overall survival was defined as the time from randomization until death. Individuals lost to follow-up prior to death were censored at the time of last physician contact. Toxicity/adverse event reported was scored using CTCAE Version 4.0.

### Exploratory correlates

We sought to determine whether treatment with CC-486 resulted in greater demethylation compared to patients monitoring with SOC observation. Paired tissue biopsies at pre-treatment baseline and time of recurrence were obtained to evaluate DNA methylation status. The methylation analysis population consisted of all subjects with any evaluable methylation results including plasma and/or tumor biopsies. Following DNA extraction, all biopsy samples were bisulfite treated and hybridized to Illumina Human Epic arrays for methylation analysis. All analysis was performed in R. Illumina Human Epic methylation array data were preprocessed using the funNorm algorithm [20] implemented in the minfi package [21] from Bioconductor, to produce beta values and detection *p* values. CpG sites on the X and Y chromosomes were excluded from subsequent analysis. Empirical Bayes Linear models as implemented in the limma package [22] from Bioconductor were used to test whether individual CpG sites showed decreased in methylation after treatment.

### Statistical methodology

#### Sample size and evaluable patients

The study was designed to enroll 60 patients (30 per arm) with PFS as the primary endpoint. This planned sample size provided 80% power to identify a 64% improvement in the median PFS from the null rate of 6 months assuming a one-sided type 1 error rate of 0.20, 24 months of accrual, and a minimum of 12 months of follow-up while allowing for 10% loss to follow-up. The intent to treat cohort included all radiographic disease-free patients who were randomized. All patients who received at least one dose of CC-486 were included in the safety analysis. Observation-only patients were not evaluated for adverse events as they did not receive the study agent.

#### Futility analysis

An interim futility analysis was planned after 50% of the expected progressions have occurred. The conditional power was calculated for the following three scenarios for the unobserved data: 1) null hypothesis—assumed no difference between the two treatment arms, i.e., the median PFS in both groups is 6 months; 2) alternative hypothesis—assumed that the originally hypothesized rates (6 months vs. 9.84 months) would be observed; and 3) current pattern—assumes that the PFS observed for each group in the initial cohort would be observed for the remaining patients. The decision to stop or continue was based upon the currently observed pattern with the null and alternative hypothesis patterns providing an estimate of the range of possible values. The trial would halt recruitment and concluded futility if the conditional power is 30% or less.

#### Endpoint analysis

Time-to-event outcomes, including the primary endpoint PFS and OS, were summarized using Kaplan–Meier estimates of the survival function. Cox proportional hazards models were used to compare the two treatment arms and to evaluate and adjust for potential prognostic factors (e.g., nodal status, microscopic margins, increase in CA19-9, previous SOC therapy). Logistic regression was used to compare the response rate between the groups and to identify risk factors associated with response. For exploratory correlative outcomes, summary statistics (e.g., means, standard errors) and plots were used to describe the pharmacodynamic endpoints at each time point (resection and relapse) as well as the change over time. *T* tests and Fisher’s exact tests were used to assess the differences in the change in pharmacodynamic outcomes between those with and without CC-486 exposure. We performed 2-sided, paired-sample, empirical Bayes moderated t tests on each CpG site evaluated on the Illumina Epic methylation array. Results were considered statistically significant for *p* values < 0.05 for clinical and pharmacodynamic analyses and for Benjamini–Hochberg adjusted *p* values < 0.1 for DNA methylation correlates.

## Results

### Patient enrollment

A total of 49 participants were enrolled and randomized. The conditional power was < 1% at the planned interim analysis, which occurred once 31 progressions (recurrence or death) were observed. Therefore, enrollment was halted prior to recruiting the planned 60 participants and the intention to treat cohort included a total of 48 evaluable participants: *n* = 25 (52%) were assigned to observation, and *n* = 23 (48%) were assigned to CC-486. One of the participants originally randomized to the CC-486 arm had progressive disease at the time of randomization and was considered non-valuable for clinical outcomes. This individual was included in all other analyses according to their assigned treatment per the analysis plan described in the protocol (Additional file 1: Study Protocol). Notably, two participants in OBS arm withdrew consent after treatment assignment and were censored on the date of randomization. Three additional participants withdrew from consent for study follow-up and were censored for death and progression at the time they withdrew from follow-up.

The baseline demographic and disease characteristics, including time from surgery to randomization, were largely well balanced between the two treatment arms (Table 1). Median time from surgery to randomization was 9.6 months (mo) (range 2.9–36.8). The proportion of individuals who received neo-adjuvant chemotherapy had R1 resections at time of surgery, and CA19-9 levels > 100 at time of enrollment were higher among those assigned to CC-486 (21%) as compared to observation (8%) (Table 1).

**Table 1:** Baseline demographic and disease characteristics of study cohort

Characteristic	Total	Observation	CC-486
	(*N* = 49)	(*N* = 25)	(*N* = 24)
Eligibility criteria, *N*(%)			
CA19-9	24 (49%)	12 (48%)	12 (50%)
R1	10 (20%)	2 (9%)	8 (33%)
Positive lymph nodes	36 (73%)	20 (80%)	16 (67%)
Age at randomization, Median (1st–3rd Q)	66 (60, 71)	64 (50, 73)	66 (61, 68)
Female, *N*(%)	23 (47%)	10 (40%)	13 (54%)
Race			
White	46 (94%)	24 (96%)	22 (92%)
Asian	2 (4%)	1 (4%)	1 (4%)
Other	1 (2%)	0 (0%)	1 (4%)
ECOG at randomization, *N*(%)			
0	40 (82%)	19 (76%)	21 (88%)
1	9 (18%)	6 (24%)	3 (12%)
CA 19–9 at randomization, Median (1st–3rd Q)	37.3 (15.4, 83.9)	37.3 (13.6, 77.8)	36.9 (18.9, 90.7)
Time from surgery to randomization (months), Median (1st–3rd Q)	9.6 (7.8, 12.0)	9.6 (7.8, 12.2)	9.8 (8.1, 10.9)
Surgical Resection Margin Status, *N*(%)			
R0	39 (80%)	23 (92%)	16 (67%)
R1	10 (20%)	2 (8%)	8 (33%)
Histologic grade, *N*(%)			
Well differentiated	3 (6%)	2 (8%)	1 (4%)
Moderately differentiated	36 (74%)	17 (68%)	19 (79%)
Poorly differentiated	10 (20%)	6 (24%)	4 (17%)
T stage, *N*(%)			
T1 (≤ 2 cm)	13 (27%)	6 (24%)	7 (29%)
T2 (> 2 cm)	16 (33%)	10 (40%)	6 (25%)
T3 (> 4 cm)	19 (39%)	9 (36%)	10 (42%)
T4 (involves celiac axis, SM or common hepatic artery)	1 (2%)	0 (0%)	1 (4%)
Positive lymph nodes, *N*(%)	36 (73%)	20 (80%)	16 (67%)
Lymphovascular invasion (LVI), *N*(%)			
No	17 (39%)	6 (27%)	11 (50%)
Yes	27 (61%)	16 (73%)	11 (50%)
Missing	5 (10%)	3 (12%)	2 (8%)
Perineural spread/invasion (PNS), *N*(%)	43 (88%)	23 (92%)	20 (83%)
Pre-trial Radiation Therapy, *N*(%)			
None	18 (37%)	10 (40%)	8 (33%)
Neo-adjuvant only	11 (22%)	3 (12%)	8 (33%)
Adjuvant only	20 (41%)	12 (48%)	8 (33%)
Pre-trial Systemic Therapy, *N*(%)			
Neo-adjuvant only	1 (2%)	1 (4%)	0 (0%)
Adjuvant only*	30 (61%)	18 (72%)	12 (50%)
Both neo-adjuvant and adjuvant	18 (37%)	6 (24%)	12 (50%)
Neo-adjuvant Systemic Therapy, *N*(%)			
None	30 (61%)	18 (72%)	12 (50%)
5-FU-based combination	15 (31%)	6 (24%)	9 (38%)
Gemcitabine-based combination	2 (4%)	0 (0%)	2 (8%)
Other	2 (4%)	1 (4%)	1 (4%)
Adjuvant Systemic Therapy, *N*(%)			
None	1 (2%)	1 (4%)	0 (0%)
5-FU-based combination	15 (31%)	4 (16%)	11 (46%)
Gemcitabine-based combination	10 (20%)	6 (24%)	4 (17%)
Gemcitabine alone	10 (20%)	6 (24%)	4 (17%)
Gemcitabine/Xeloda	9 (18%)	5 (20%)	4 (17%)
Other	4 (8%)	3 (12%)	1 (4%)

*One individual received a single dose of gemcitabine

*N* Number; % percent; *Q* Quartile

### Clinical efficacy

A total of 48 patients (*n* = 25 OBS, *n* = 23 CC-486) were included in the intention to treat (IIT) cohort analysis. With a median follow-up time of 20.3 months (IQR 12.8, 41.4), a total of 34 (*n* = 18 CC-486, *n* = 16 OBS) confirmed progressions were observed (Table 2). Confirmed radiographic recurrence sites included 16 patients with locoregional recurrence only, 9 with distant only, 8 with both locoregional and distant progression, and 1 death. Four additional patients had clinical progression (e.g., functional deterioration combined with rising CA19-9 levels without clear imaging correlate).

**Table 2:** Progression-free and overall survival analysis (from time of study randomization)

Outcome	Observation	CC-486
Confirmed progression-free survival		
*N* at risk	25	23^**a**^
*N* events	16	18
Median (95% CI)	8.9 (3.4, 24.6)	9.2 (4.1, 20.9)
1-year PFS, % (95% CI)	50% (27%, 69%)	38% (18%, 58%)
Hazard ratio (95% CI)		0.94 (0.46, 1.87)
* P* value		*P* = 0.85
Progression-free survival including clinical assessments		
*N* at risk	25	23^a^
*N* events	19	19
Median (95% CI)	6.2 (3.2, 18.7)	9.17 (2.5, 20.9)
1-year PFS, % (95% CI)	43% (23%, 63%)	37% (17%, 56%)
Hazard ratio (95% CI)		0.83 (0.42, 1.59)
* P* value		*P* = 0.57
Overall survival		
*N* at risk	25	24
*N* events	14	15
Median (95% CI)	26.4 (17.8, 46.7)	33.8 (12.8, 47.6)
2-year OS, % (95% CI)	61% (36%, 78%)	50% (28%, 69%)
Hazard ratio		0.98 (0.46, 2.05)
* P* value		*P* = 0.96

^a^One patient randomized to CC-486 was found to have progressive disease prior to start of treatment and was thus not included in the PFS analyses

**Fig. 2:**
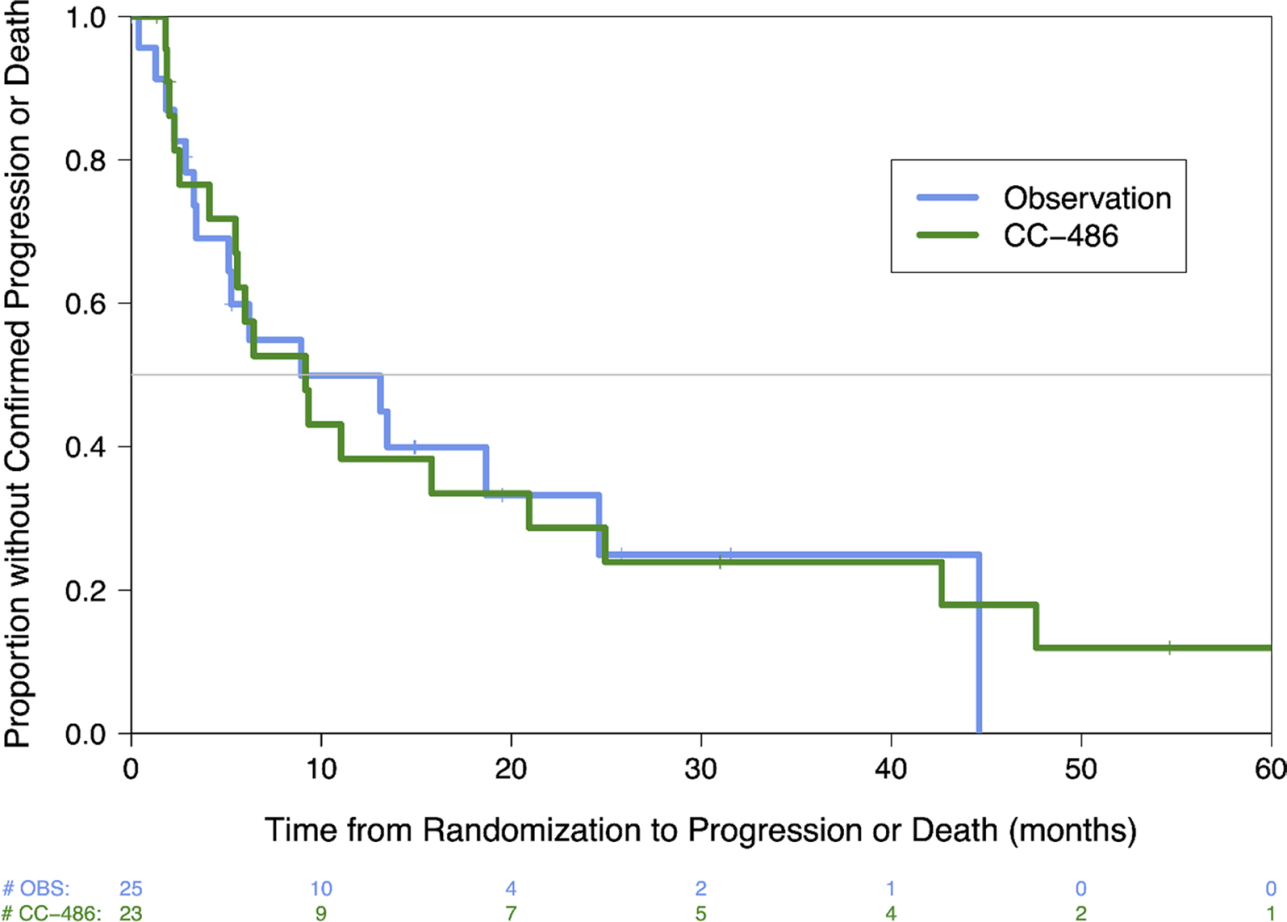
Kaplan–Meier estimate of the progression-free survival for participants assigned to receive observation (blue) or CC-486 (green). Individuals were considered to have progressed if they had progression confirmed by radiography or biopsy or died

**Fig. 3:**
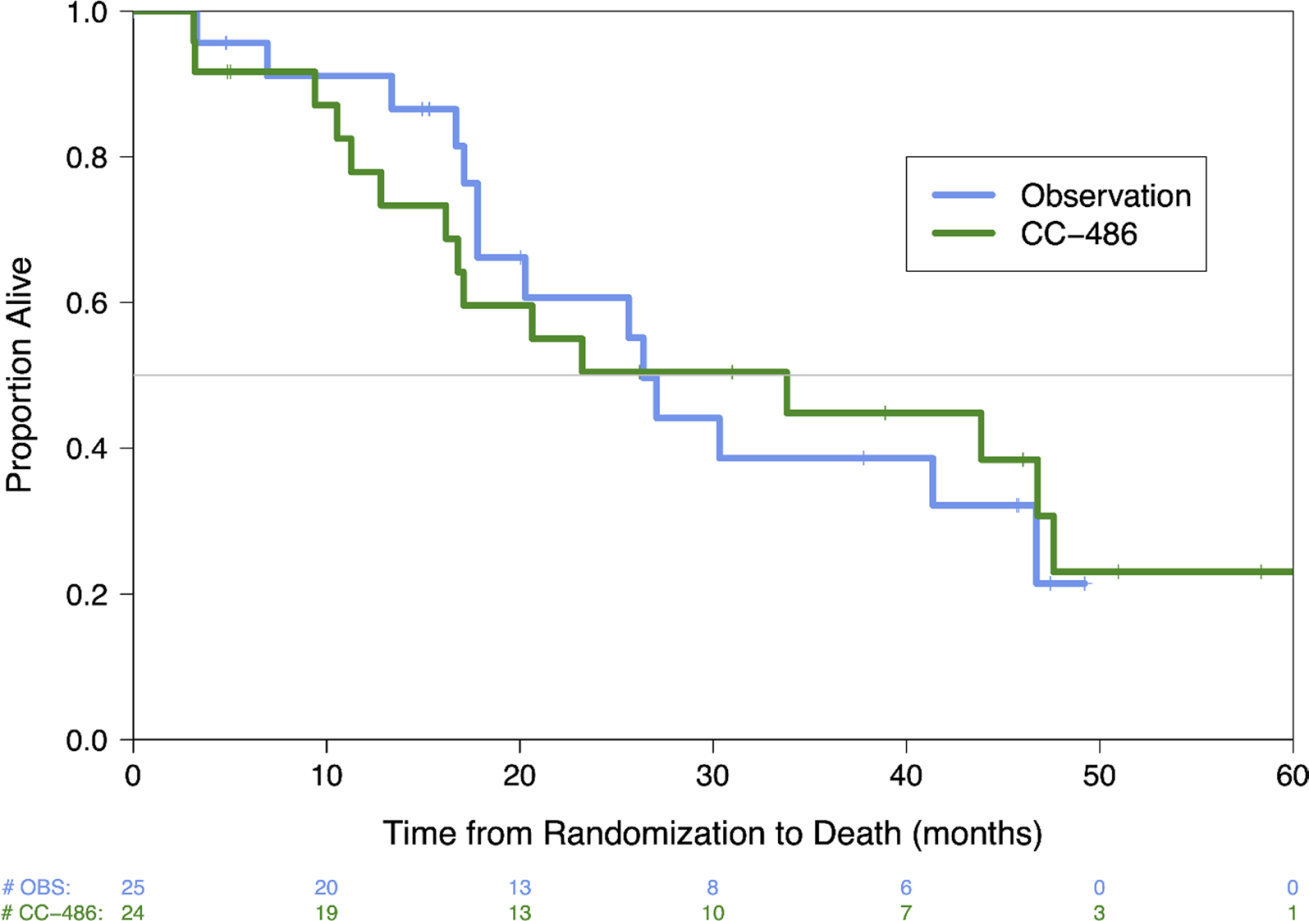
Kaplan–Meier estimate of the overall survival for participants assigned to receive observation (blue) or CC-486 (green)

Median PFS (1-yr PFS%) in patients with documented radiographic progression was 9.2 (38%) and 8.9 months (50%) for CC-486 and OBS arms, respectively, with no significant difference (HR 0.94, 95% CI 0.46–1.87, *p* = 0.85) between the arms (Fig. 2, Table 2). When factoring in clinical progressions, median PFS to 6.2 mo for the OBS arm but the differences between the arms did not reach statistically significance (Table 2). CA 19–9 of 100 or higher at the start of the trial was associated with a significant increase in risk of confirmed progression (HR 7.73 [2.51 – 23.77] *p* < 0.001) (Additional file 2: Table S1). No other risk factors were significantly associated with PFS (Additional file 2: Table S1). Median OS (2-yr OS%) was 33.8 (50%) and 26.4 months (61%) in CC-486 and OBS, respectively, with no significant difference (HR 0.98, 95% CI 0.46–2.05, *p* = 0.96) between the arms (Fig. 3, Table 2).

### Clinical outcomes with subsequent first-line chemotherapy (metastatic setting)

Of the 38 participants with radiographic or clinically suspected recurrence, 25 (*n* = 12 CC-486, *n* = 13 OBS) went on to receive chemotherapy in the advanced disease/metastatic setting and were evaluable for outcomes (ORR and metastatic PFS [MPFS]) (Additional file 2: Table S2). The median follow-up time from the start of metastatic chemotherapy was 5.9 months (range 1.8–29.2). The median duration of metastatic therapy was 3.5 months (range 1.1–35.1) including two participants that were still on therapy at the time of the data freeze (Additional file 2: Table S2).

Participants assigned to CC-486 were significantly more likely to have received adjuvant 5-FU-based combination therapy as compared to those assigned to observation (62% vs. 14%, *p* = 0.018) and were less likely to receive 5-FU-based combination therapy during metastatic treatment (23% vs. 77%, *p* = 0.017) (Additional file 2: Table S3). At the time of metastatic chemotherapy initiation, 53% of CC-486 patients had distant metastasis at time of start of next-line therapy compared to 23% OBS patients (Additional file 2: Table S3). Additionally, all CC-486 patients evaluable for metastatic chemotherapy outcomes had radiographically confirmed recurrence prior to start of next-line therapy, while 23% (*n* = 3) of the OBS cohort did not have definitive radiographic recurrence prior to start of next-line systemic therapy (Additional file 2: Table S3). The ORR to next-line systemic therapy was similarly minimal in both cohorts (8% for CC-486, 0% for OBS, *p* = 0.99). MPFS on next-line systemic therapy was 9.1 [6-mo MPFS 69%) and 5.0 (6-mo MPFS 33%) for OBS and CC-486 arms, respectively (Additional file 2: Tables S3 and S4). While this [MPFS] was significantly lower among patients previously treated with maintenance CC-486 compared to patients previously in the OBS arm (HR 2.49, 95 1.00–6.18, P = 0.049), the overall survival for the two groups did not differ significantly.

### Safety/Toxicity

Twenty-three out of 24 participants assigned to the CC-486 arm received at least one dose of study therapy and were included in the safety analysis. The median treatment duration was 5.6 mo (range 1.3–12.8). A total of 115 treatment-related toxicities were observed in 23 participants with 14 grade 3–4 toxicities occurring in 5 individuals (2, 2, 1 individuals had 2, 3, 4, respectively) (Table 3). The most common mild grade AEs were nausea, fatigue, and diarrhea. Grade 3 events included diarrhea and cytopenias. All grade 4 events were due to neutropenia or leukopenia. Five (22%) patients required a dose reduction, 7 (30%) patients experienced dose-delays, and 4 (17%) patients discontinued therapy due to drug toxicity. There were no study drug-related serious adverse events observed.

**Table 3:** Adverse event and grading for individuals in the CC-486 arm that received at least one dose of medication

Adverse Event Type	Grade 1 # events (# patients)	Grade 2# events (# patients)	Grade 3# events (# patients)	Grade 4# events (# patients)	Any Grade# events (# patients)
*Summary for each grade*					
Counts	76 (23)	25 (12)	9 (4)	5 (3)	115 (23)
Events/month*	0.49	0.16	0.06	0.03	0.75
*Summary by AE type*					
Constitutional					
Appetite/weight loss	12 (11)	0	0	0	12 (11)
Dizziness	3 (3)	0	0	0	3 (3)
Fatigue	11 (10)	6 (6)	0	0	17 (13)
Headache	1 (1)	0	0	0	1 (1)
Gastrointestinal			0	0	
Abdominal Discomfort/Bloating	3 (3)	1 (1)	0	0	4 (4)
Constipation	2 (2)	1 (1)	0	0	3 (3)
Diarrhea	12 (10)	1 (1)	2 (1)	0	15 (10)
Nausea	17 (12)	4 (2)	0	0	21 (13)
Dyspepsia	1 (1)	0	0	0	1 (1)
Flatulence	3 (3)	0	0	0	3 (3)
Vomiting	2 (2)	2 (2)	0	0	4 (3)
Hematologic					
Anemia	3 (3)	2 (2)	2 (1)	0	7 (5)
Leukopenia	1 (1)	4 (4)	3 (3)	1 (1)	9 (6)
Lymphocytopenia	0 (0)	0 (1)	1 (1)	0 (0)	1 (1)
Neutropenia	0	2 (2)	1 (1)	4 (3)	7 (5)
Thrombocytopenia	1 (1)	2 (1)	0	0	3 (1)
Other					
Fever	1 (1)	0	0	0	1 (1)
Hyperglycemia	1 (1)	0	0	0	1 (1)
Muscle cramps	1 (1)	0	0	0	1 (1)
Oral abscess	1 (1)	0	0	0	1 (1)

*Rate per month is based upon a total follow-up of 153.7 months across all patients

### Tumor tissue methylation analysis

Five patients (*n* = 3 [CC-486], *n* = 2 [OBS]) had paired pre-treatment baseline and recurrence biopsies available for analysis. Only 3 of these paired samples (*n* = 2 [CC-486], *n* = 1 [OBS]) had sufficient quantities of viable input DNA to successfully run DNA methylation arrays. The majority of sites (66%) showed a decrease in methylation after treatment with CC-486; however, because of the limited number of samples, treatment-induced changes in the CpG site methylation did not reach statistical significance (Additional file 2: Fig S1).

## Discussion

This is the first clinical trial to assess the role of maintenance epigenetic therapy in the resected PDA population. Treatment with CC-486 following standard adjuvant therapy did not improve progression-free survival in patients with high-risk rPDA nor improve response to next-line chemotherapy or overall survival compared to standard observation following adjuvant treatment. The near 80% observed recurrence rate illustrates the vulnerability of this target patient population and the need for effective maintenance therapy following standard adjuvant therapy.

The hypothesis behind this study was that as epigenetic mechanisms are known to be pivotally involved in the acquired ability of malignant cells to metastasize, that the utilization of epigenetic modulators would be effective in delaying or eliminating metastatic disease after the completion of adjuvant therapy. However, the observed median PFS in this trial from start of maintenance therapy compares similarly to previous published retrospective cohort studies assessing the role of maintenance chemotherapy in resected PDA patient following standard adjuvant therapy [23–25]. The lack of benefit of maintenance CC-486 is unfortunately consistent with previously published early phase trials evaluating DNMT inhibitors (alone or in combination with other epigenetic therapies such as HDAC inhibitors) showing minimal to no efficacy solid tumors treated in the advanced stage [26–31].

As we probe these data, the baseline risk of recurrence between the groups may not have been equal. While largely balanced, on post hoc review, the two groups appeared to have some notable differences. Specifically, the CC-486 group close to three times the number of participants with CA19-9 levels > 100 at time of enrollment compared to those randomized to the observation arm. CA19-9 levels > 100 at time of enrollment was strongly associated with worse PFS. Study therapy patients also were more likely have received neo-adjuvant systemic therapy (50% vs. 28%), usually utilized in cases with more negative clinical prognostic features on imaging including suspicious lymphadenopathy, borderline resectable disease technically, or elevated tumor markers. Finally, the experimental cohort had a higher frequency of positive margins on surgical resection (*n* = 8 [33%] vs. *n* = 2 [8%]). Taken together, the baseline risk of recurrence at time of randomization may have been higher in the experimental cohort.

While this trial also hypothesized a possible beneficial priming effect of epigenetic therapy on next-line systemic chemotherapy responses, the small numbers and variability among patterns of disease recurrence and chemotherapy regimens utilized (both in terms of systemic agents and incorporation of radiation therapy) limited the interpretation of these results. More generally, an added challenge to the interpretation of the results of this study is that patients received a heterogeneous mix of adjuvant and or neo-adjuvant treatment regimes in the perioperative setting, including treatment timing, agents used, and incorporation or not of radiation.

One of the important lessons from this trial was the challenging pace of patient accrual with less than 50 patients enrolled over the 7 years the trial was open. Our institution is a high-volume center with over 175 pancreatic cancer surgeries a year. The study team prescreened over 500 patients over the course of the trial. Yet, the focus of this study on high-risk resected patients resulted in many patients progressing prior to being eligible for the study, including multiple screen-fails at the time of study entry for progressive disease. These data highlight a challenging reality of the significant unmet need for new therapies even in this subset of resected patients who had surgery for curative intent.

Finally, correlative methylation analysis was limited by the number viable serial tissue biopsies available. Our findings are consistent with the hypothesis that CC-486 reached the tumor and changed the methylation landscape, but, with only 2 samples, the evidence is very limited. A larger number of paired tissue biopsies are needed to better assess the pharmacodynamics and onsite drug action of these medications.

While the role of single-agent epigenetic therapy remains limited in PDA, the efficacy of concurrent administration with other non-redundant epigenetic agents, chemotherapies, and/or immunotherapies remains an important area for further clinical inquiry. Furthermore, stratifying patients with biomarker/genetic/epigenetic profiling may allow for more optimal patient selection for future epigenetic agents and treatment strategies.

## Conclusion and future directions

Treatment with CC-486 following adjuvant therapy did not prolong time to relapse in patients with high-risk resected PDA nor improve overall survival. The high proportion of recurrent disease illustrates the vulnerability of this target population and the challenge to find effective maintenance therapy following standard adjuvant therapy for patients with resected pancreatic cancer. With the minimal clinical efficacy demonstrated in solid tumor treatment thus far, the role of epigenetic therapy will be dependent on novel epigenetic agents, clinical experience with concurrent administration with standard and immunotherapies, and precision-medicine strategies using robust predictive biomarkers for patient selection in future clinical trials [32].

## Supplementary Information

**Additional file 1**. Study Protocol.

**Additional file 2**. Supplemental Materials. **Fig S1:** Global change in methylation after treatment. [a] The following violin plots show change in methylation after treatment, compared to before. Change is calculated as the simple difference between methylation levels after and before treatment. [b] The relationship between FDR and change in beta is shown in the following volcano plot. The red lines indicate a change in beta of 0.10. **Table S1:** Association between confirmed progression free survival and characteristics of interest. **Table S2:** Characteristics of patients received metastatic chemotherapy stratified by study treatment group. **Table S3:** Best Response to Systemic Therapy in Advanced Disease Setting. **Table S4:** Association between metastatic progression free survival and characteristics among participants receiving chemotherapy in the advanced disease setting.

## Data Availability

The datasets used and/or analyzed during the current study are available from the corresponding author upon reasonable request and IRB approval, as indicated.
