# Supplementary material for: A randomized, phase II trial of oral azacitidine (CC-486) in patients with resected pancreatic adenocarcinoma at high risk for recurrence
Source: Clin Epigenetics. 2022 Dec 3;14:166. doi: 10.1186/s13148-022-01367-8 (PMC9719150; doi:10.1186/s13148-022-01367-8)
Supplement: Supplementary file 1 — Additional file 1. Study Protocol. [file 13148_2022_1367_MOESM1_ESM.pdf]

SKCCC J12138
IND 117362

Title: A Phase II trial to improve outcomes in patients with resected pancreatic adenocarcinoma at high risk for recurrence using epigenetic therapy

**IND Sponsor:**	**Nilofer Azad, MD**
**Study drug provided by:**	Celgene Corporation<br>86 Morris Ave<br>Summit, NJ 07901<br>1-800-742-3107
**Coordinating Center**:	Johns Hopkins Sidney Kimmel Comprehensive Cancer Center
**Principal Investigator**:	Nilofer Azad, MD<br>Sidney Kimmel Comprehensive Cancer Center at Johns Hopkins<br>1650 Orleans Street, CRB1, Room 4M10<br>Baltimore, Maryland 21287<br>Phone: 410-614-9169<br>Fax: 410-614-9006<br>Email: nazad2@jhmi.edu
**Study Coordinator**:	Tearra Miles<br>201 N. Broadway<br>Baltimore, MD 21287<br>Phone: 410-502-5358
**Research Nurse:**	Sheila Linden, RN<br>550 North Broadway, Suite 415<br>Baltimore, MD 21205<br>Phone: 410-614-4397
**Statisticians**:	Elizabeth Sugar, PhD<br>615 North Wolfe St., W5010<br>Baltimore, MD 21205<br>Phone: 410-502-3919<br>Fax: 410-502-4621

Participating Sites

Peter J. O'Dwyer, M.D.
The Hospital of the University of Pennsylvania
16 Penn Tower 3400 Spruce St.
Philadelphia, PA 19104
Peter.O'Dwyer@uphs.upenn.edu
Office: 215.662.7606
Fax: 215.349.8551

Dulabh K. Monga, M.D.
Allegheny General Hospital
320 East North Ave
Pittsburgh, PA, 15212
412-359-8924 (P)
412-442-2198 (F)
dmonga@wpahs.org

CONFIDENTIAL
The information contained in this document is regarded as confidential and, except to the extent necessary to obtain informed consent, may not be disclosed to another party unless law or regulations require such disclosure. Persons to whom the information is disclosed must be informed that the information is confidential and may not be further disclosed by them.

Confidential

PRINCIPAL INVESTIGATOR SIGNATURE PAGE

Principal Investigator/Sponsor Approval:

This clinical study protocol has been reviewed and approved by the study representative listed below:

Nilofer Azad, MD	
**Print Name**	**Signature**
Associate Professor, Johns Hopkins	
**Title, Medical Institution**	**Date (DD MMM YYYY)**

Investigator's Signature:

I have fully discussed the objectives of this study and the contents of this protocol with the Sponsor's representative.

I understand that information contained in or pertaining to this protocol is confidential and should not be disclosed, other than to those directly involved in the execution or the ethical review of the study, without written authorization from the Sponsor. It is, however, permissible to provide information to a subject in order to obtain consent.

I agree to conduct this study according to this protocol and to comply with its requirements, subject to ethical and safety considerations and guidelines, and to conduct the study in accordance with the Declaration of Helsinki, International Conference on Harmonization guidelines on Good Clinical Practice (ICH E6), and applicable regional regulatory requirements.

I agree to make available to Sponsor personnel, their representatives and relevant regulatory authorities, my subjects' study records in order to verify the data that I have entered into the case report forms. I am aware of my responsibilities as a Principal Investigator as provided by the Sponsor.

I understand that the Sponsor may decide to suspend or prematurely terminate the study at any time for whatever reason; such a decision will be communicated to me in writing. Conversely, should I decide to withdraw from execution of the study, I will communicate my intention immediately in writing to the Sponsor.

Confidential

----------------------------- --

Print Name **Signature**

------------------------- ---

Title **Date (DD MMM YYYY)**

Table of Content

1 Protocol Synopsis 9
1.1 Description 9
1.2 Schema 12
2 Schedule of Study Assessments 13
2.1 Schedule during Initial Phase – Arm A & Arm B 13
2.2 Schedule during Follow Up – All Subjects* 16
3 Background and Rationale 17
3.1 Adenocarcinoma of the pancreas 17
3.2 Epigenetic modification in cancer 19
3.3 5-Azacitidine 19
3.3.1 Indication and Usage 22
3.3.2 Adverse Events 22
3.4 Rationale for Treatment in this Setting 23
3.5 Correlative Studies 24
4 Study Objectives and Endpoints 25
4.1 Objectives 25
4.2 Endpoints 25
5 Investigational Plan 26
5.1 Overall design 26
5.1.1 5-Azacitidine Description 26
5.2 Screening and Eligibility 29
5.2.1 Inclusion Criteria 29
5.2.2 Exclusion criteria 30
5.2.3 Inclusion of Women and Minorities 31
5.2.4 Patient Recruitment 31
5.2.5 Protocol and Drug Administration 31
5.2.6 Multicenter Guidelines 31
5.2.7 Patient Registration 32
5.2.8 Randomization 33

5.2.9 Dosage Dispensing and Administration 33
5.2.10 Record of administration 34
5.3 Visit Schedule, Assessments, and Dose Modifications 34
5.3.1 Screening and Pre-Study Assessments 35
5.3.2 Initial Phase: Assessments, Dose Modifications, and Termination 35
5.4 Discontinuation of Treatment: Arm A 38
5.4.1 End of Treatment 39
5.5 Follow-up Phase Both Arms 39
5.6 Concomitant Therapy 40
5.6.1 Recommended Concomitant Therapy 40
5.6.2 Prohibited Concomitant Therapy 41
5.7 Treatment adherence 41
6 Adverse events 41
6.1 Definitions Adverse Event (AE) Definition 41
6.1.1 Serious Adverse Event (SAE) Definition 41
6.2 Adverse Event Reporting 42
6.2.1 Pregnancies 44
6.2.2 Celgene Drug Safety Contact Information: 45
6.3 Investigator Reporting Responsibilities 45
6.3.1 Expedited reporting by investigator to Celgene 45
6.3.2 Report of Adverse Events to the Institutional Review Board 45
6.3.3 Investigator Reporting to the FDA 45
6.4 Adverse event updates/IND safety reports 46
7 Radiologic Evaluation 46
7.1 Baseline imaging assessments 46
7.2 Definitions of Measurable and Non-Measurable Disease 47
7.3 Guidelines for Evaluation of Measurable Disease 48
7.3.1 Measurement Methods 48
7.3.2 Acceptable Imaging Modalities for Measurable Disease 48

7.3.4 Measurement of Effect 49
7.3.5 Response Criteria during Follow-up Phase 49
7.3.6 Overall Objective Status during Follow-up Phase 50
8 Correlative Studies 52
8.1 Plasma/Serum Samples 52
8.2 Tumor Biopsies 53
8.2.1 Specimen Collection and Handling 54
9 Protocol Amendments/Deviations 55
9.1 Protocol Amendments 55
9.2 Protocol Deviations 55
10 Data Management 55
10.1 Data Collection 55
10.2 Analyses and Reporting 55
10.3 Clinical Trial Monitoring 56
10.4 Investigator Responsibilities 56
10.5 Safety Meetings 57
11 Biostatistical Analysis 57
11.1 Study Design/Endpoints 57
11.2 Study Populations 57
11.2.1 Efficacy Evaluable Population 57
11.2.2 Methylation Analysis Population 57
11.3 Statistical Methodology 58
11.3.1 Efficacy Analyses 58
11.3.2 Methylation Analyses 58
11.3.3 Quality of life analysis 58
11.3.4 Sample Size 59
11.3.5 Safety Monitoring 59
11.3.6 Futility Analysis 60
12 Regulatory Considerations 61

12.1 Institutional Review Board/Ethics Committee approval 61
12.2 Informed Consent 62
12.3 Subject Confidentiality 62
12.4 Study Records Requirements 62
12.5 Premature Discontinuation of Study 62
13 References 63
14 Appendices 67
Appendix A: ECOG Performance Status Scale 67
Appendix B: Medication Diary 68
Appendix C: Recommendations for Management of Treatment-Induced Diarrhea (for CC-486) 70
Appendix D: FACT – Hep (Version 4) 71

1 Protocol Synopsis

1.1 Description

**PROTOCOL TITLE:** A phase II trial to improve outcomes in patients with resected pancreatic adenocarcinoma at high risk for recurrence using epigenetic therapy	
**DATE PROTOCOL FINAL:**	
**INDICATION:**	Cancer Therapy
**STUDY PHASE:**	Phase II

BACKGROUND AND RATIONALE:

DNA methylation is often abnormal in cancer cells and can lead to the inactivation of tumor suppressor genes. Hypomethylating agents, such as 5-azacitidine, have been shown to reverse DNA hypermethylation and allow for reactivation of tumor suppressor genes and ultimately lead to cancer cell death. These agents are currently the standard of care in myelodysplasia and have also shown some efficacy in early phase trials for solid tumors. In pancreatic cancer cell lines, promoter regions of several tumor suppressor genes have been demonstrated to be abnormally hypermethylated. The addition of hypomethylating agents has restored the expression of these genes and has decreased tumor growth. While most of the trials have used the subcutaneous delivery of azacitidine, the oral formulation, CC-486, is currently being studied in a phase I trial for MDS/AML and has been shown to be bioavailable, well-tolerated, and produce similar clinical responses as the subcutaneous version.

Pancreatic adenocarcinoma is a very deadly disease, with only 20% of patients being candidates for possible curative surgery at presentation. However, the majority of these patients will recur. Patients that are node positive or margin positive at the time of resection have a greater than 90% chance of recurrence and death from disease. After surgery and adjuvant therapy, patients are monitored with CT scans and CA 19-9 levels. In some patients, CA 19-9 levels rise before visible disease progression, suggesting micro-metastatic disease. After completing adjuvant chemotherapy, further chemotherapy is typically not used before recurrent disease is visible even with an elevated biomarker; however a therapy that is well-tolerated would be ideal in this setting. The goal of this trial is to demonstrate that CC-486 is well tolerated and effective when given after adjuvant therapy in patients with high risk disease (rising CA 19-9, node positive disease, or margin positive disease) by prolonging time to visible disease recurrence, increasing response rates to eventual chemotherapy, and increasing overall survival.

STUDY OBJECTIVES:

Primary objective:

To improve progression free survival in high risk patients with resected pancreatic adenocarcinoma who have node positive disease, margin positive disease, and/or elevation in CA 19-9 treated with CC-486 as compared to observation after completion of adjuvant therapy.

Secondary objectives:

1) To improve response rates to first-line chemotherapy (partial and complete response) after recurrence in patients treated with CC-486 after completing adjuvant therapy.
2) To improve overall survival in patients with resected pancreatic adenocarcinoma treated with CC-486.
3) To evaluate resected pancreatic cancer tissue and biopsies at time of recurrence for epigenetic and genetic alterations to determine the pharmacodynamic effects of CC-486.
4) To evaluate resected pancreatic cancer tissue and blood samples to identify predictive signatures of possible recurrence and the benefit of hypomethylating therapy.
5) To evaluate quality of life during CC-486 using the FACIT-Hep questionnaire

STUDY ENDPOINTS:

Primary endpoint:

The primary endpoint of the trial is to demonstrate an increase in the progression free survival, PFS, in resected pancreatic cancer patients treated with CC-486.

Secondary endpoints:

1) Response rate (including partial and complete response) to first-line chemotherapy when given after visible disease recurrence in patients primed with CC-486 compared to observation.
2) Overall survival
3) Decreased methylation in candidate genes in the research biopsies as compared to the original resected specimens in patients treated with CC-486.
4) Change in quality of life as determined by FACIT-HEp scores

STUDY DESIGN:

This trial is for patients with resected pancreatic adenocarcinoma who have concluded adjuvant therapy or were deemed unable to receive adjuvant therapy with an elevated CA 19-9 or node positive or margin positive disease. CA 19-9 elevation is defined as two levels > the institutional upper limit of normal (ULN) taken at least 2 weeks apart. These levels should be measured after adjuvant therapy has concluded or upon the decision that adjuvant therapy will not be offered. Patients will be randomized to one of two arms. Subjects enrolled due to node + disease or R1 resection must be able to undergo randomization within 3 months of finishing adjuvant therapy or the decision that they are unable to take adjuvant therapy. Patients enrolling due to CA 19-9 elevation can enroll any time after adjuvant therapy has completed. Group A, the treatment arm, will be started on CC-486. Group B, the control arm, will receive no additional therapy. In both arms, CA 19-9 will be followed and CT scans (or MRI, if clinically indicated) will be done every three months. When patients have visible disease recurrence on imaging, CC-486 will be stopped and both groups will start first-line chemotherapy.

STUDY DURATION:

Assuming the maximum accrual with a goal of 2-3 subjects accrued per month, we estimate enrollment duration of 24 months with an additional 12 months for follow-up with concurrent data analysis and manuscript preparation.

TOTAL SAMPLE SIZE:

Maximum 60 patients, with 30 patients in each of the two arms.

DRUG SUPPLIES:

For study participants, Celgene Corporation will provide CC-486

DOSING REGIMEN:

Treatment Arm	***DRUG***	***DOSE***	***FREQUENCY***	***# of CYCLES***	***Route***
**A**	CC-486	300 mg total, three 100 mg tablets	Daily, Days 1-21, Q28 Day cycles	12 cycles and then follow-up	Oral
**B**	Observation	N/A	N/A	12 cycles. Follow-up 1 will be collected 3 months after Cycle 10.	N/A

1.2 Schema

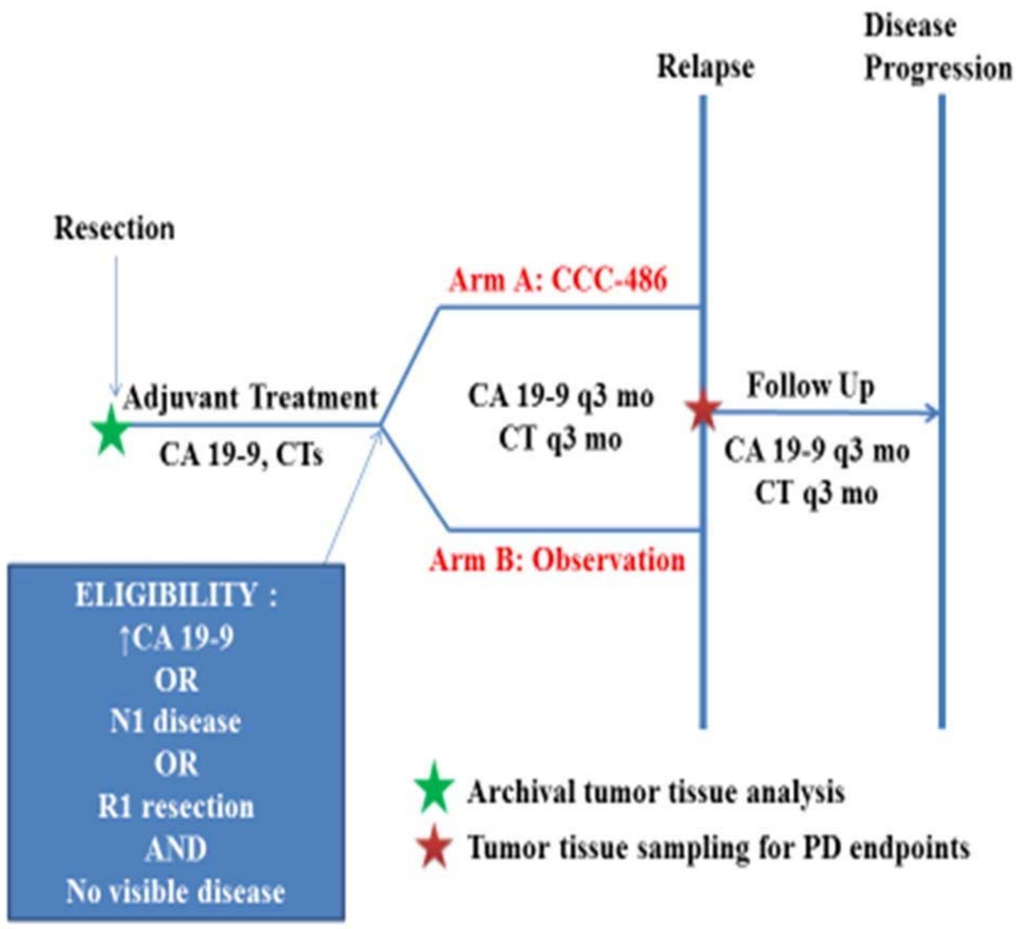
Disease Progression
Relapse
Resection
Arm A: CCC-486
Adjuvant Treatment
CA 19-9, CTs
CA 19-9 q3 mo
CT q3 mo
Follow Up
CA 19-9 q3 mo
CT q3 mo
Arm B: Observation
ELIGIBILITY :
↑CA 19-9
OR
N1 disease
OR
R1 resection
AND
No visible disease
Archival tumor tissue analysis
Tumor tissue sampling for PD endpoints

2 Schedule of Study Assessments

2.1 Schedule during Initial Phase – Arm A & Arm B

For Arm A ±3 day window for study assessments and ± 2 weeks for Tumor Assessments.
For Arm B, schedule variations are permitted for Tumor Assessments and CA19-9 collection

Procedure	Screening	Cycle 1		Cycles 2, 3, 5, 6, 8, 9, 11 and 12	Cycles 4, 7 and 10		Safety Assessment	Upon Visible Disease Progression
	≤28 days prior to C1D1	(Day 1)		(Day 1)	(Day 1)		*28 days post d/c CC-486*	
		Arm A	Arm B	Arm A	Arm A	Arm B	Arm A	Arm A & B
CC-486 dosing (D1-21)[20]		X		X	X			
Prior medications & anti-cancer Therapies	X							
Physical examination	X	X[1]		X	X		X	X[7]
Vital signs	X	X[1]		X	X		X	X[7]
Weight	X	X[1]		X	X		X	X[7]
ECOG performance status[9]	X	X[1]		X	X		X	X[7]
Tumor assessment including CT or MRI chest/abd/pelvis[10]	X				X[11]	X[6, 11, 15]		X[7]
CBC with differential	X	X[1]		X	X		X	X[7]
Serum chemistry and hepatic panel	X	X[1]		X	X		X	X[7]
PT/PTT	X							X[7]
CA 19-9	X	X[1]	X[1, 6]		X	X[6, 18]		X[7]
Pregnancy Test[12]	X	X[1]						

Procedure	Screening	Cycle 1		Cycles 2, 3, 5, 6, 8, 9, 11 and 12	Cycles 4, 7 and 10		Safety Assessment	Upon Visible Disease Progression
	≤28 days prior to C1D1	(Day 1)		(Day 1)	(Day 1)		*28 days post d/c CC-486*	
		Arm A	Arm B	Arm A	Arm A	Arm B	Arm A	Arm A & B
Blood collection for correlative studies[19]	X	X[1, 3]	X[1, 2, 4]	X[3]	X[3]	X[2, 4]		X[8]
Tumor biopsy[13, 19]								X
Record adverse events/symptoms[17]	X	X[1]		X	X		X	X
Collect remaining pills and diary[21]				X	X		X	X
Record concomitant therapies/procedures	X	X[1]		X	X		X	X
Archival Tissue[16, 19]	X							
Quality of Life	X	X[1]	X[1, 5]		X	X[5]	X[14]	X[7]

In order to minimize the need for research-only in-person visits, telemedicine visits may be substituted for in person clinical trial visits or portions of clinical trial visits where determined to be appropriate and where determined by the investigator not to increase the participants risks. Prior to initiating telemedicine for study visits the study team will explain to the participant, what a telemedicine visit entails and confirm that the study participant is in agreement and able to proceed with this method. Telemedicine acknowledgement will be obtained in accordance with the Guidance for Use of Telemedicine in Research. In the event telemedicine is not deemed feasible, the study visit will proceed as an in-person visit. Telemedicine visits will be conducted using HIPAA compliant method approved by the Health System and within licensing restrictions.

1 If study assessment assessed within 7 days of Cycle 1, Day 1, they do not need to be repeated.

2 Correlative blood sampling is optional for patients in Arm B except for at screening and at time of visible disease progression.

3 For patients assigned to Arm A, research blood will be collected for tumor methylation analysis and other correlative studies on Day 1 of *every* cycle.

4 For patients assigned to Arm B, research blood will be collected for tumor methylation analysis and other correlative studies at screening and/or Cycle 1 Day 1 and Day 1 of *every third* cycle beginning with Cycle 4 (Cycle 4, 7, 10).

[5] Patients on Arm B can provide this information to study team member over the phone.

[6] Patients on Arm B who opt not to participate in correlative blood sampling may be assessed by their local oncologist for all clinical, laboratory and imaging parameters.

[7] If study assessment was completed within 7 days of noting visible disease progression, they do not need to be repeated.

[8] For Arm A patients, if research blood was collected within one month, it does not need to be repeated. For Arm B patients, if research blood was collected within 3 months, it does not need to be repeated.

[9] Please refer to Appendix A for definition of ECOG performance status.

[10] Radiologic imaging of chest/abdomen/pelvis (CT preferred, abdominal/pelvis MRI will be performed for patients with CT contrast allergy) and any other imaging required per RECIST for the particular subject are required at baseline must be obtained within 4 weeks of initiation of therapy.

[11] Assessment for presence of visible disease will occur prior to every third cycle, beginning with Cycle 4 (Cycles 4, 7, 10). For each individual patient, the same imaging modality should be used at assessment as was used during screening.

[12] Obtain a urine or serum pregnancy test at screening no more than 7 days prior to dosing on Day 1.

[13] Tumor biopsies will be obtained at the time of visible disease recurrence for all subjects. Location of biopsy can be primary tumor or site of metastatic disease, as determined by the treating oncologist. Tumor biopsy may be obtained within 4 weeks of visible disease progression or before first dose of chemotherapy, whichever occurs latest.

[14] Completed only if FACIT data not collected at time of visible disease progression

[15] Tumor assessments should ideally be performed within a +/- 2 week window every 3 months (Cycle, 4, 7, and 10). However, Tumor assessment may be completed at the patient's oncologist's discretion outside of the ideal window

[16] May be obtained any time throughout the study for both Arm A and Arm B patients.

[17] Assessment not required for Arm B patients

[18] For Arm B patients CA19-9 may be collected outside of the recommended 3 month window at the patient's local oncologist's discretion.

[19] Research samples will be collected at the discretion of the PI based on availability of supplies and safety of patient and staff.

[20] CC-486 dosing (D1-21) research drug may be shipped to patients to limit contact due to COVID-19 precautions. See **Section 5.2.9.1** for more details.

[21] Review of remaining pills and pill diary may be reviewed during a telemedicine visit when drug is unable to be returned to study staff due to COVID-19 precautions. Copies of the pill diary may be submitted to study staff by phone, email, fax, or mychart.

2.2 Schedule during Follow Up – All Subjects*

Assessments are to be completed within 3 months of safety assessment/progression/previous follow-up

Schedule variations for CA19-9 and Imaging assessments permitted

**Procedure** *	**Follow Up (Until 2nd disease progression)**	**Survival Follow-Up** (≤ 3mo of previous follow up)**
Information on new cancer therapies (records to be obtained)	X	
Subjective response to therapies (records to be obtained)	X	
CA 19-9 (records to be obtained)	X	
Imaging completed (records to be obtained)	X	
Study team contact***	X	X

* For Arm B patients, follow up will be completed within 3 months of cycle 10 or within 3 months of disease progression. For Arm A patients, follow up will be completed within 3 months of safety assessment. If safety assessment was not completed, follow up will begin within 3 months of progression/discontinuation of drug. Follow Up assessments are ideally to be completed on a 3 month schedule, in which subsequent follow ups will be completed within 3 months of the previous follow-up. However, CA.19 and Imaging may be completed per the patient oncologist's discretion. An attempt to contact patients and obtain information on new cancer therapies and subjective responses to such therapies will occur within 3 months of the previous follow up.

** Survival Follow-Up will begin once the patient is deemed to have progressive disease for the second time. If the patient has not progressed and was put on Follow Up for reasons other than visible disease progression, patient must still have two instances of visible disease progression until being on the Survival Follow Up schedule. Patients will remain on the selected Follow-Up schedule for a combined total of up to 4 years, death or the subject withdraws consent (whichever occurs first).

*** Study team will attempt to contact the patient in order to obtain information regarding his/her survival status, and any other comments/information per PI's request. While it is preferred the study team contacts the patient for follow-up, this information may also be obtained through medical records and obituaries.

3 Background and Rationale

3.1 Adenocarcinoma of the pancreas

Pancreatic adenocarcinoma has one of the highest incidence-to-mortality ratios of any disease. Although it represents the 10th leading cause of cancer in the US, it is the 4th leading cause of cancer-related deaths. In 2011, there was an estimated 44,030 new cases and 37,660 deaths from pancreatic cancer in the US alone[1]. At diagnosis, at most 20% of patients are surgical candidates and thus potentially curable, however the majority will recur and eventually die of their disease. Their median survival is still under 2 years with a 20% 5-year survival[2-4]. A standard adjuvant treatment approach for patients with resected disease has not yet been determined, although chemotherapy or chemoradiation therapy is most commonly used. Patients that have positive lymph nodes (negative nodes is N0, positive nodes is N1) or positive margins (negative margin is R0, microscopic is R1) at the time of their resection have a very high risk of developing recurrence as their cancer has already spread beyond the pancreas itself. In the event of tumor recurrence, defined as visible disease present on imaging, further chemotherapy is given. However, an earlier sign of recurrence is sometimes a rising blood CA 19-9 level, a tumor marker indicative of presence of disease, without visible tumor seen on imaging. Our goal is to give these patients who are high risk of recurrence (N1 disease, R1 margins, or elevated CA 19-9) a therapeutic option before their disease reveals itself on imaging. Epigenetic agents are drugs that function by reactivating tumor suppressor genes to slow cancer progression. This project aims to slow the recurrence of pancreatic cancer in these high risk patients by treatment with the epigenetic drug, azacitidine.

Positive lymph nodes found at the time of resection have been consistently shown to be a poor prognostic factor for overall survival in resected pancreatic adenocarcinoma[5]. As a result of this, lymph node status is incorporated into the AJCC staging classification. Patients with positive nodes regardless of being T1, T2 or T3 disease are stage IIB[6]. It is recommended that a minimum of 12-15 nodes should be removed during a pancreaticoduodenectomy procedure (Whipple) in order to accurately stage a patient as node positive or node negative. This number was shown to be critical when 1150 patients who had undergone a Whipple procedure were examined from the SEER database. Patients that had N0 disease when 15 or more lymph nodes were removed had an 8 month improved survival as compared to the patients who had N0 disease when less than 15 nodes were removed[7]. Similarly, patients who had N0 disease with fewer than 12 nodes removed had a similar survival to those with a few positive nodes in another retrospective review[8]. This implies that the patients with fewer than 12-15 removed lymph nodes may have occult positive nodal disease that is not found on pathologic review and could lead to a worse to a worse survival than expected based on stage. However, examining more than 15 nodes does not improve the survival based on a prospective trial randomizing patients to standard Whipple versus extended

lymphadenectomy. In both the standard and the extended group, the patients with node positive disease had about an 11-19% decrease in survival at 1 year and 21-44% decrease at 3 years when compared to the node negative patients and there was no difference in survival among the surgery groups[9]. In a retrospective review of 1423 patients who underwent Whipple procedures from 1970 to 2006 at The Johns Hopkins Hospital, 78% had positive lymph nodes. On multivariate analysis, positive lymph node status was a predictor for shorter survival[10]. Nodal involvement was also shown to be an independent predictor of worse outcome in an analysis of a phase II Japanese trial of neoadjuvant therapy[11], an MD Anderson retrospective review[12], as well as in the RTOG 9704 trial where the hazard ratio for N0 disease was 0.63 when compared to patients with 1-3 positive nodes[13]. A large meta-analysis again showed that median survival across all studies of N0 patients was 25 months as compared to 13.6 months for all of the N1 patients[14].

The relationship between positive margins and worse survival has not been as conclusively determined.[5] Both the Johns Hopkins Hospital[10] and the MD Anderson[12] reviews demonstrated that positive resection margins were independent predictors of decreased survival. The large meta-analysis also demonstrated an increase in survival from 10.3 months for all patients with R1 resections compared to 20.3 months for patients with R0 resections[14]. The benefit of an R0 resection was proven to improve overall survival despite a larger surgery when a retrospective review compared patients who had pancreaticoduodenectomy with a positive pancreatic neck margin with patients who were converted to a total pancreatectomy and had negative margins. Median overall survival was 10 months in the R1 group versus 18 months in the R0 group[15]. However, margin status wasn't a significant factor in a multivariate analysis in a retrospective study of 291 patients after taking into account size and stage of the tumor[16].

Serum biomarkers have been evaluated to aid in the early detection of pancreatic cancer and CA 19-9 has shown the most promise to help differentiate carcinoma from non-malignancy[2, 3]. It can also be used in the peri-resection state where higher levels are associated with increased tumor size, higher stage, and shorter survival[17]. The RTOG 9704 trial evaluated patients who received adjuvant chemoradiation after resection and those with post-op CA 19-9 ≥ 180 had a median survival of 9 months compared to 21 months when CA 19-9 < 180[18]. Patients who undergo resection and adjuvant therapy are monitored with imaging and CA 19-9 levels, but are not commonly offered more treatment until disease recurrence is evident on imaging given the cumulative toxicities of standard chemotherapy. However, rising CA 19-9 levels are likely an early sign of local recurrence or micro-metastatic disease and a treatment that can be easily tolerated would be beneficial in order to lengthen time to visible disease progression and survival. Although there is no published data, our experience is that it usually takes between 3 and 6 months for patients to develop visible disease after their CA 19-9 rises. We are currently performing a retrospective study of patients who have undergone surgery at Johns Hopkins and continue to be followed here to further clarify this observation. Our preliminary results show that only 35% of

patients have a normal CA 19-9 after surgery. 50% have an elevated level with the remainder having no documented level after surgery. Of those with normal post-surgery levels, 20% have CA19-9 rise prior to visible disease with a lag time of 1.5 – 7.5 months. The remainder either develops visible disease first or at the same time.

3.2 Epigenetic modification in cancer

Epigenetic modification is the process by which gene expression is altered by mechanisms other than changes in the actual DNA sequence. DNA methylation, a type of epigenetic modification, occurs when DNA methyltransferases transfer a methyl group onto the 5' carbon of the cytosine pyrimidine ring of promoter regions of genes. These promoter regions are characterized by a high number of cytosine-guanine dinucleotides, called CpG islands. When the CpG islands are unmethylated, this leads to an open chromatin state that allows for the transcription of the gene that follows this promoter region. Conversely, when the CpG islands are hypermethylated, the chromatin is closed and transcription of the gene is suppressed. Therefore, hypermethylated promoter regions silence the gene which follows. In the normal cell, DNA methylation occurs in varying amounts throughout the genome in order to control gene expression. This process is often abnormal in cancer cells[19-21].

Tumor suppressor genes are a large category of genes that control cell proliferation through various mechanisms like regulating the cell cycle, repairing damaged DNA, and encouraging apoptosis[22]. They act as the stop signals for cells. However, when these genes are inactivated, cell growth is not properly controlled and damaged cells can accumulate and lead to tumorgenesis. In normal cells, the promoter regions for these tumor suppressor genes are unmethylated thereby allowing for transcription of the genes. In cancer cells, however, some of these promoter regions are hypermethylated leading to inactivation and silencing of the tumor suppressor gene[20, 23]. This mechanism is a very intriguing target for cancer therapy as DNA methylation, like other epigenetic modification, is reversible[20, 23].

3.3 5-Azacitidine

5-Azacitidine (AZA) and 5-aza-2'-deoxycytidine (decitabine, DAC) are pyrimidine nucleoside analogs of cytidine, both with 5' modifications of the ring. These drugs were initially used at high doses in the 1970's as cytotoxic agents, mainly in hematologic malignancies. They have since been shown to promote DNA hypomethylation at lower doses by incorporating themselves in DNA and RNA, irreversibly binding to DNA methyltransferases, and causing decreased methylation in daughter cells. This allows for reactivation of tumor suppressor genes leading to cessation of cancer cell growth, cancer cell damage and ultimately cancer cell death[24]. In myelodysplastic syndrome and in elderly patients with acute myelogenous leukemia, AZA and DAC have demonstrated improved overall survival and time to leukemia progression[25-27]. A

phase III trial randomized 358 patients with high-risk MDS to receive either AZA or best supportive care. Overall survival was 24.5 months in the AZA group compared to 15.0 months in the control group[28]. AZA and DAC are now considered the standard of care for patients with intermediate or high-risk MDS and elderly patients with AML who cannot receive high intensity treatment[29]. As these agents work best in cells as they are diving, optimal efficacy has been seen after several months as the drugs get incorporated into the cells[24] and therefore are likely most effective in situations when time is available.

While this phase III data was done using a subcutaneous administration of azacitidine, the oral formulation, CC-486, (typically given for 7-21 days and repeated every 28 day cycle) is currently being studied in early phase trials in this patient population. It was first shown to be safe at a low dose in a pilot pharmacokinetic study in 2008[30]. In the phase I study supported by Celgene to determine the maximum tolerated dose of CC-486, 41 patients with MDS, CMML or AML refractory or inappropriate for conventional therapies received one cycle of subcutaneous azacitidine followed by cycles of CC-486 given days 1-7 of every 28 day cycle. The maximum tolerated dose (MTD) was 480 mg. The dose limiting toxicity was severe diarrhea, experienced in 2 of 3 of the patients at the 600 mg dose. Nausea, vomiting, headache, fatigue, fever, cough and febrile neutropenia were the other grade 3 adverse events observed. 8 patients were still receiving the study drug at the time of the paper's publication. CC-486 was rapidly absorbed with a mean bioavailability at the MTD of 13%. CC-486 did cause hypomethylated of a significant number of loci with peak effect at day 15 of each cycle, although not to the same extent as subcutaneous azacitidine. Clinical responses were seen in 35% of patients with MDS and CMML who were previously treated and in 73% of patients who received this as first-line therapy. Therefore, CC-486 has proved to be bioavailable, well tolerated and produce similar clinical response as the subcutaneous version[31]. This group also presented more detailed pharmacokinetic and pharmacodynamic data at the 11th International Symposium on Myelodysplastic Syndrome in Scotland in May 2011 of the above subcutaneous followed by oral dosing as well as oral dosing starting from the first cycle. This poster demonstrated data from patients who in 28 day cycles took CC-486 200 mg days 1-14, 200 mg days 1-21, 300 mg days 1-14, and 300 mg days 1-21. The 300 mg given days 1-21 of a 28 day cycle demonstrated reduced DNA methylation, provided 56% of the cumulative exposure to 5azacitidine as compared to 7 days of the subcutaneous version, and was well tolerated.

The impact of methylation and use of hypomethylating agents has also been studied in solid tumors. In lung cancer, there have been suggestions of efficacy, with some heavily pretreated metastatic patients showing tumor shrinkage or disappearance, and other patients that have lived longer than expected even without discrete tumor shrinkage. Decitabine has been shown to act in nasopharyngeal carcinoma and gastric adenocarcinoma to allow for the re-expression of the tumor suppressor gene TSLC1[32] and ID4[33], respectively. Epigenetic therapy is presently being tested in

colon and breast cancer as well. Most of the studies used subcutaneous azacitidine, however some recent phase I trials are investigating the use of the oral formulation, CC-486. In December 2011, a Phase Ib study of CC-486 (AZA-ST-001) began enrolling subjects with relapsed or refractory solid tumors. In the safety study, patient were assigned at the investigator's discretion to either Arm A (escalating doses of CC-486 given days 1-14 of a 21 day cycle in combination with carboplatin AUC 4 on day 8 of a 21 day cycle), Arm B (escalating doses of CC-486 given days 1-14 of a 21 day cycle in combination with *nab*paclitaxel 100 mg/m2 on days 8 and 15 of a 21 day cycle), or Arm C (escalating doses of CC486 on days 1-21 of a 21 day cycle). About 18 patients have completely at least one cycle of treatment. There have been 4 DLTs to date at the 200 mg dose level of CC-486: 2 on Arm B, 1 on Arm 1 and 1 on Arm C. All 4 DLTs were neutropenia of which 2 were febrile neutropenia (arm B and arm C) and 2 were delayed to start of cycle 2 (arm B and arm A). Arm B has since been amended to eliminate the day 21 dose of *nab*-paclitaxel. The study is continuing to enroll and a MTD has not yet been defined.

In pancreatic cancer, hypermethylation of the promoter regions of mismatch repair genes (*hMLHI*)[34], growth inhibitory genes (*ARHI*)[35], cell cycle control genes (*Cyclin D2*)[36], and proapoptotic genes (*TNFRSF10c*[37], *TMS1*[38], and *CRABP2*[39]) have been shown to lead to the loss of expression of those genes which contributes to tumor growth. When DAC was added to pancreatic cancer cells that exhibited hypermethylation of the gene *ARHI*, *ARHI* was reexpressed, cell growth was inhibited by almost 50%, and apoptosis was increased by 10-fold. Pancreatic cancer cells were then implanted into mouse xenografts, DAC was added to half of the cohort, and the mice were sacrificed after 3, 5, 7, or 9 days. As compared to the control mice, those that were treated with DAC had a decrease in the volume of their tumor by 52% after 3 days of treatment and by 92% after 9 days, demonstrating that the addition of a hypomethylating agent can slow the growth of the tumor[35]. Pancreatic cancer cell lines with hypermethylation of *TNFRSF10c* also showed an increase in gene expression by up to 8 times, a decrease in promoter region methylation by over 50%, as well as an increase in apoptosis with the addition of DAC[37]. When AZA was added to pancreatic cancer cells with hypermethylated *TMS1*, there was a 2.5 fold increase in *TMS1* expression. Furthermore, when gemcitabine or docetaxel were added to the cells, the ones that were pre-treated with AZA had a 25-30% increase in cell death after the chemotherapy as compared to the cells without pre-treatment[38]. Lack of expression of CRABP2 in pancreatic cell lines has been shown to make them resistant to treatment with retinoic acid. The promoter regions were found to be hypermethylated in these resistant cell lines and expression of CRABP2 was increased with the treatment of DAC. As CRABP2 over expression is known to cause apoptosis in other types of tumor cell lines, increasing its expression may make these cells more sensitive to treatment including retinoic acid[39]. More recently, a putative tumor suppressor gene, myeloid/lymphoid or mixed-lineage leukemia 3 (MLL3) has been shown to have diminished expression in pancreatic tumor cell lines, tumor xenografts and archived patient

tumors. Upon further lowering the MLL3 expression by RNAi, these cell lines demonstrated increased transformation and invasion under in vitro conditions. Its CpG island promoter region was found to be hypermethylated. Treatment with a DNA methyltransferase inhibitor caused hypomethylation and re-expression of the MLL3 gene in the cell lines. Therefore, hypomethylating agents that allow for these genes to be re-activated can differentiate between cancerous and non-cancerous tissues and control tumor growth. In addition, hypermethylation of these promoter regions may cause disruptions of the apoptotic pathway that contribute to the chemotherapy resistance seen in pancreatic cancers. This data supports our hypothesis that pretreating patients with CC-486 will impair tumor growth and increase their sensitivity to chemotherapy.

3.3.1 Indication and Usage

5-Azacitidine is indicated for treatment of patients with both low and high-risk myelodysplastic syndrome (MDS) for the following French-American-British (FAB) subtypes: refractory anemia (RA) or refractory anemia with ringed sideroblasts (RARS) (if accompanied by neutropenia or thrombocytopenia or requiring transfusions), refractory anemia with excess blasts (RAEB), refractory anemia with excess blasts in transformation (RAEB-T), and chronic myelomonocytic leukemia (CMMoL). It is also considered a category 2B recommendation per the NCCN guidelines (based on lower-level evidence with a non-uniform NCCN consensus but no major disagreement) for elderly patients with AML who cannot receive conventional high-intensity chemotherapy.

3.3.2 Adverse Events

The most common adverse reactions (>30%) for the subcutaneous route per the package insert are: nausea, anemia, thrombocytopenia, vomiting, pyrexia, leukopenia, diarrhea, injection site erythema, constipation, neutropenia and ecchymosis. The most common adverse reactions by IV route also include petechiae, rigors, weakness and hypokalemia.

The most common adverse reactions for the oral route (CC-486) per the phase I trial were: diarrhea, nausea, constipation, vomiting, abdominal pain, headache, fatigue, peripheral edema, fever, cough, confusion, dizziness and febrile neutropenia. The DLT was diarrhea[31].

Per Celgene’s experience, very common side effects (10% or more patients experienced these) include: nausea, vomiting; neutropenic fever; inflammation of the nasal membranes or nose and/or throat pain; chest pain; dizziness; headache; rash; bruising; and decreased appetite.

Common side effects (affected 1-10% of patients) included: anemia; thrombocytopenia; neutropenia with or without fever; infections such as pneumonia, urinary tract infection, skin

infection, and blood infection with or without hypotension; inflamed pouches in the lining of the intestine; indigestion; abdominal pain; diarrhea; fever; loss of fluid; heart failure; heart and lungs stop working; bleeding including bleeding in individual organs; hypokalemia; decreased consciousness, with drowsiness, listlessness and apathy; feeling unwell; anxiety; insomnia; hair loss; redness of the skin; serious allergic reaction to a blood transfusion; joint, bone, or muscle pain; shortness of breath with or without exercise; orthostatic hypotension; and seizure. Uncommon side effects (affected 0.1-1% of patients) included: constipation; bowel obstruction; swelling in the bowel; sore mouth; fluid, thickening, inflammation, or scarring in the lungs; pulmonary embolism; difficulty breathing; irregular heartbeats; inflamed or swollen heart; feeling tired or weak; worsening of general condition/disease; weight loss; fainting; confusion; balance problems; depression; chills; abnormal blood pressure; failure of the bone marrow to make enough blood cells; cytopenias; splenomegaly; gall bladder problems; kidney failure; allergic reaction; flank pain; neck or extremity pain; and itching or skin infection causing open sores, itchy rash, skin swelling, or collection of pus on an arm or leg; and other cancers. Tumor lysis syndrome was also noted.

Complete and updated adverse events are available in the Investigational Drug Brochure and the IND Safety Letters.

3.4 Rationale for Treatment in this Setting

Pancreatic cancer is a highly aggressive malignancy. Increasing preclinical and clinical evidence has suggested that epigenetic therapy requires several months to achieve optimal efficacy, creating a great challenge in a rapidly progressing cancer. High-risk pancreatic cancer patients with positive nodal disease, positive resection margins, and/or elevated CA 19-9, are a unique patient population to test this type of treatment. The window of time until disease progression provides an ideal opportunity to employ epigenetic therapy. Currently, there is no consensus as to how one should treat patients with pancreatic cancer who have undergone resection and adjuvant therapy but have an elevated CA 19-9. Although CA 19-9 has a variable specificity for identifying pancreatic adenocarcinoma, its continual rise in a patient with previously known disease highly suggests microscopic recurrence, despite a lack of visible disease on scans. Physicians hesitate to give conventional cytotoxic chemotherapy in the absence of clear disease; however patients and physicians are left unsettled given the knowledge that the disease is recurring. Similarly, patients who have N1 disease or R1 resections are not typically given any further treatment after completing their adjuvant therapy in the absence of disease; however virtually all of these patients will recur. Azacitidine is a very well tolerated therapy that can be given to patients in this time period without significant toxicity. Preclinical models demonstrate that multiple tumor suppressor genes have abnormally hypermethylated promoter regions in pancreatic cancer, therefore AZA would allow for these genes to be reactivated and to slow tumor

growth. Furthermore, there is preclinical and clinical data in other solid tumors that suggests that AZA may prime the cancer to improve its response to chemotherapy – a hypothesis that our study will also test.

3.5 Correlative Studies

Correlative studies will be performed to determine if therapy with CC-486 results in demethylation which can be detected in tumor tissues, as well as plasma. These studies will also evaluate for an epigenetic signature that can help predict patients who will be responders versus non-responders and identify predictive signatures of possible recurrence and benefits of CC-486 therapy.

Blood for correlative studies will be drawn from both Arm A and Arm B patients at screening and/or on Day 1 of Cycle 1. Patients in Arm A will have research blood work done on Cycle 1 Day 1 if Day 1 is not within 7 days of screening, and Day 1 of every subsequent cycle.

For patients in Arm B, additional research blood work is optional, except for at visible disease progression. If patients in Arm B choose to provide research blood, it will be collected every third cycle beginning at Cycle 4. Research blood will be collected at recurrence if it was not collected within the last three months.

These correlative studies are done in order to evaluate the methylation status of free tumor DNA circulating in the blood. Quantitative analysis using real-time Methylation-specific PCR (MSP) will be used to detect the presence of methylated alleles for specific genes. Candidate genes found to be hypermethylated in pre-treatment samples will be monitored for changes in methylation during and after treatment using real-time MSP. Methylation status analysis will also be done on pre-treatment and post-treatment samples using a novel nano-enabled methylation based technology, Methylation-on-Beads. This assay has shown great promise as a tool for surveillance of responses to epigenetic therapy. Blood will also be stored to use in the future if new biomarkers are developed that may better predict recurrence.

All patients will also undergo a tumor biopsy at the time of their recurrence on scans that will be examined with their original resected pancreatic tumor. All biopsy specimens will undergo tumor methylation analysis with MSP for select candidate genes (*TMS1*, *ARH1*, etc). Where sufficient tissue is obtained, samples will also be studied using HumanMethylation 450K BeadChip (Illumina). This technology examines over 450,000 CpGs and we will analyze the data and integrate the methylome data with mRNA or miRNA expression array studies.

4 Study Objectives and Endpoints

4.1 Objectives

Primary objective:

To improve progression free survival in patients with resected pancreatic adenocarcinoma who have node positive disease, margin positive disease, and/or elevation in CA 19-9 treated with CC-486 as compared to observation after completion of adjuvant therapy.

Secondary objectives:

1) To improve response rates to first line chemotherapy (partial and complete response) after recurrence in patients treated with CC-486 after completing adjuvant therapy

2) To improve overall survival in patients with resected pancreatic adenocarcinoma treated with CC-486.

3) To evaluate resected pancreatic cancer tissue and biopsies at time of recurrence for epigenetic and genetic alterations to determine the pharmacodynamic effects of CC-486.

4) To evaluate resected pancreatic cancer tissue and blood samples to identify predictive signatures of possible recurrence and the benefit of hypomethylating therapy.

5) To evaluate quality of life on CC-486 using the FACIT-Hep questionnaire

4.2 Endpoints

Primary endpoint

The primary endpoint of the trial is to demonstrate an increase in the progression free survival, PFS, in resected pancreatic cancer patients treated with CC-486

Secondary endpoints

1) Response rate (including partial and complete response) to first line chemotherapy when given after visible disease recurrence in patients primed with CC-486 compared to observation

2) Overall survival

3) Decreased methylation in candidate genes in the research biopsies as compared to the original resected specimens in patients treated with CC-486.

4) Change in quality of life scores as determined by FACIT-Hep

5 Investigational Plan

5.1 Overall design

This is a randomized, phase II study of CC-486 versus observation in patients with resected pancreatic adenocarcinoma who have finished adjuvant chemotherapy and/or chemoradiation (or were deemed unable to receive adjuvant therapy) with no evidence of visible disease on imaging and have high risk features. High risk features are defined as an elevated CA 19-9 level > ULN drawn on 2 occasions at least 2 weeks apart; positive nodal disease; and/or positive resection margins. The study will be conducted at Johns Hopkins University, The University of Pennsylvania, Massachusetts General Hospital, and Beth Israel Deaconess Medical Center. Patients will be randomized to one of two arms to enable us to evaluate for effects of CC-486 in this population. Arm A will be started on CC-486 at 300 mg daily for 21 days, repeated every 28 day cycle. The maximum tolerated dose from the phase I trial in patients with MDS, CMML and AML was 480 mg given for 7 days every cycle[31], however this lower dose given for a prolonged period of time appeared to be tolerated better and had good bioavailability. Arm B will receive observation only. It will be recommended that patients obtain a CA 19-9 and tumor assessment every three months, however, schedule variations are permitted at the oncologist's discretion. Patients on the observation arm who are unable to travel to study site may be followed by their local oncologist ideally adhering to the same schedule. When patients have visible disease recurrence on CT scan, Arm A will stop the CC-486 and may begin their preferred cancer treatment regime. Similarly, after visible recurrence on imaging, Arm B will begin their preferred cancer treatment regime. Both Arm A and Arm B patients will follow the Follow-up schedule after their first visible disease progression. Once patients have had a 2nd instance of visible disease progression, they will be moved to the survival follow-up schedule. We will power this study in order to see a 64% increase of the progression free survival between the two groups. This will require approximately 60 patients to be enrolled and randomized.

5.1.1 5-Azacitidine Description

5-Azacitidine is a pyrimidine nucleoside analog of cytidine. 5-Azacitidine is 4-amino-1-β-Dribofuranosyl-s-triazin-2(1H)-one. The empirical formula is $C_8H_{12}N_4O_5$. The molecular weight is 244. The structural formula is:

Chemical Structure of 5-Azacitidine

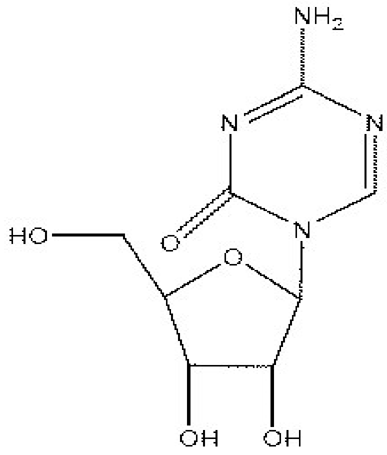

5-Azacitidine is a white to off-white solid. It was found to be insoluble in acetone, ethanol, and methyl ethyl ketone; slightly soluble in ethanol/water (50/50), propylene glycol, and polyethylene glycol; sparingly soluble in water, water saturated octanol, 5% dextrose in water, N-methyl-2-pyrrolidone, normal saline and 5% Tween 80 in water; and soluble in dimethylsulfoxide (DMSO).

5.1.1.1 Clinical Pharmacology

5-Azacitidine is a pyrimidine nucleoside analog of cytidine. It is believed to exert its antineoplastic effects by causing hypomethylation of DNA and direct cytotoxicity on abnormal hematopoietic cells in the bone marrow. The concentration of 5-azacitidine required for maximum inhibition of DNA methylation *in vitro* does not cause major suppression of DNA synthesis. Hypomethylation may restore normal function to genes that are critical for differentiation and proliferation. The cytotoxic effects of 5-azacitidine cause the death of rapidly dividing cells, including cancer cells that are no longer responsive to normal growth control mechanisms. Non-proliferating cells are relatively insensitive to 5-azacitidine.

5.1.1.2 Pharmacokinetics

The pharmacokinetics of 5-azacitidine were studied in 6 MDS patients following a single 75 mg/m^2 subcutaneous (SC) dose and a single 75 mg/m^2 intravenous (IV) dose. 5-Azacitidine is rapidly absorbed after SC administration; the peak plasma 5-azacitidine concentration of 750 ± 403 ng/ml occurred in 0.5 hour. The bioavailability of SC 5-azacitidine relative to IV 5azacitidine is approximately 89%, based on area under the curve. Mean volume of distribution following IV dosing is 76 ± 26 L. Mean apparent SC clearance is 167 ± 49 L/hour and mean half-life after SC administration is 41 ± 8 minutes.

Published studies indicate that urinary excretion is the primary route of elimination of 5azacitidine and its metabolites. Following IV administration of radioactive 5-azacitidine to 5 cancer patients,

the cumulative urinary excretion was 85% of the radioactive dose. Fecal excretion accounted for <1% of administered radioactivity over 3 days. Mean excretion of radioactivity in urine following SC administration of 14C-5-azacitidine was 50%. The mean elimination half-lives of total radioactivity (5-azacitidine and its metabolites) were similar after IV and SC administrations, about 4 hours.

The pharmacokinetics of CC-486 as a single agent were evaluated in the phase I/II trial in MDS, CMML and AML patients. Plasma and urine studies were done on days 1 and 7 of cycle 1 (SQ dosing) and cycle 2 (oral dosing). Rapid absorption was seen in both routes as the maximum observed plasma concentration (C_{max}) was reached within 0.5 hours with SQ dosing and 1.0 hours with oral dosing. The mean elimination half-life was 1.6 ± 0.7 hours for SC and 0.62 ± 0.25 hours for oral and the concentration decreased in a pseudobiphasic manner. The MTD had a mean relative bioavailability of 13% ± 9%. Apparent volume of distribution was greater than total body water and apparent total clearance was greater than hepatic blood flow, suggestive wide tissue distribution and extrahepatic metabolism, respectively. Less than 2% of azacitidine was recovered in urine in both SQ and oral forms, implying the main elimination pathway is not renal. Tests after multiple doses suggest no azacitidine accumulation[31].

5.1.1.3 Handling, Storage, and Disposal of CC-486

CC-486 is a cytotoxic drug and, as with other potentially toxic compounds, caution should be exercised when handling CC-486 tablets. Procedures for proper handling and disposal of anticancer drugs should be applied.

If CC-486 comes into contact with the skin, immediately and thoroughly wash with soap and water. If it comes into contact with mucous membranes, flush thoroughly with water.

CC-486 will be supplied as 100 mg tablets. Each tablet is formulated using excipients that are generally regarded as safe and are used in marketed drug products. Further details can be found in the CC-486 Investigator's Brochure. There is no need to protect CC-486 from exposure to light.

Each tablet will be sealed in a blister package. CC-486 tablets should be stored at 25ºC (77ºF), excursions permitted to 15ºC to 30ºC (59ºF to 86ºF), and free of environmental extremes. The storage area should be secure and have limited access. Shelf-life evaluations of the intact blister packages, pouches, and HDPE bottles are ongoing. The CC-486 study drug will be monitored for stability for the duration of the study.

5.2 Screening and Eligibility

The Investigator is responsible for keeping a record of all subjects who sign an Informed Consent Form for entry into the study. All subjects will be screened for eligibility. Screening procedures are outlined in Section 2, Schedule of Study Assessments and unless otherwise specified, must take place within 28 days prior to initiation of therapy.

5.2.1 Inclusion Criteria

Subjects must meet the following inclusion criteria to be eligible for the study:

1. Understand and voluntarily sign an informed consent form.
2. Age ≥18 years at the time of signing the informed consent form.
3. Able to adhere to the study visit schedule and other protocol requirements.
4. Subjects must have a histologically confirmed pancreatic adenocarcinoma that has had an R0 (negative margins) or R1 (microscopically positive margins) resection.
5. Subjects must have finished adjuvant therapy, which can include chemotherapy and/or chemoradiation therapy or have been determined to be unable to take adjuvant therapy. Although patients will be expected to complete chemoradiation or chemotherapy per physician recommendations, patients who are unable to complete chemotherapy ± radiation therapy secondary to dose limiting toxicities will be eligible provided they meet study criteria.
6. Subjects enrolled due to node + disease or R1 resection must be able to undergo randomization within 3 months of finishing adjuvant therapy or the decision that they are unable to take adjuvant therapy. Patients enrolling due to CA 19-9 elevations can enroll any time after adjuvant therapy has completed.
7. All previous cancer therapy including radiation, chemotherapy, and surgery, must have been discontinued at least 4 weeks prior to treatment in this study
8. Subjects must either have a CA 19-9 value > the institutional ULN on two separate checks at least 2 weeks apart OR have had an R1 resection margin OR N1 nodal disease regardless of CA 19-9 level
9. Subjects must be free of visible disease on imaging (CT, PETCT or MRI) evaluating chest, abdomen, and pelvis within 28 days of enrollment on the study.
10. Life expectancy of greater than 12 weeks
11. ECOG performance status of ≥ 1 at study entry (see Appendix A).
12. Subjects must have normal organ and marrow function as defined below:

- Absolute neutrophil count ≥ 1500/mm³
- Leukocytes ≥ 3,000/mm³
- Platelet count ≥ 100,000/mm³
- Hemoglobin > 9.0 g/dL
- Serum creatinine levels ≤ 2 mg/dL
- Serum bilirubin levels ≤1.5 mg/dL
- AST (SGOT) and ALT (SGPT) ≤ 2.5 x ULN
- Alkaline phosphatase ≤ 2.5 x ULN

13. Free of prior malignancies for ≥ 5 years with exception of currently treated basal cell, squamous cell carcinoma of the skin, or carcinoma *in situ* of the cervix or breast.

14. Women of childbearing potential should be advised to avoid becoming pregnant and men should be advised to not father a child while receiving treatment with CC-486 or *nab*-paclitaxel. All men and women of childbearing potential must use effective methods of birth control throughout the study and for three months after completing treatment.

15. Women of childbearing potential must have a negative serum or urine β-hCG pregnancy test at screening.

16. Subjects must have < Grade 2 pre-existing peripheral neuropathy (per CTCAE)

5.2.2 Exclusion criteria

Subjects who meet any of the following exclusion criteria are not eligible for the study:

1. Any serious medical condition, laboratory abnormality, or psychiatric illness that would prevent the subject from signing the informed consent form.
2. Pregnant or breastfeeding women.
3. Use of any other chemotherapy, radiotherapy, or experimental drug or therapy within 4 weeks (6 weeks for nitrosoureas or mitomycin C) prior to enrollment on study or those who have not recovered from adverse events ≥ grade 1 due to agents administered more than 4 weeks earlier except for stable grade 2 neuropathy.
4. Subjects may not receive any other concomitant investigational agents.
5. Known or suspected hypersensitivity to 5-azacitidine or mannitol

6. Known positive for HIV or infectious hepatitis, type B or C. HIV patients are at increased risk of lethal infections when treated with marrow-suppressive therapy.
7. Uncontrolled intercurrent illness including, but not limited to, ongoing or active infection, symptomatic congestive heart failure, unstable angina pectoris, cardiac arrhythmia, or psychiatric illness/social situations that would limit compliance with study requirements.
8. Any known gastrointestinal disorders which would preclude oral administration of 5azacitidine.

5.2.3 Inclusion of Women and Minorities
Both men and women and members of all races and ethnic groups are eligible for this trial.

5.2.4 Patient Recruitment
Patients will be contacted by phone via physician referrals and from investigator attendance at the weekly Pancreatic Multidisciplinary Conference. In addition, the schedule of Whipple surgeries will be requested on a weekly basis from the operating room schedulers. Patients eligible for the study will then be contacted by phone by the investigator.

5.2.5 Protocol and Drug Administration
This study will be conducted in accordance with the Sidney Kimmel Comprehensive Cancer Center's Coordinating Center Protocol.

5.2.6 Multicenter Guidelines
<u>Protocol Chair</u>

The Protocol Chair is responsible for performing the following tasks:

- Coordinating, developing, submitting, and obtaining approval for the protocol as well as its subsequent amendments.
- Assuring that all participating institutions are using the correct version of the protocol.
- Taking responsibility for the overall conduct of the study at all participating institutions and for monitoring the progress of the study.
- Reviewing and ensuring reporting of Serious Adverse Events (SAE) Reviewing data from all sites.

<u>Coordinating Center</u>

The Coordinating Center is responsible for performing the following tasks:

- Ensuring that IRB approval has been obtained at each participating site prior to the first patient registration at that site, and maintaining copies of IRB approvals from each site.

- Managing central patient registration.
- Collecting and compiling data from each site.
- Establishing procedures for documentation, reporting, and submitting of AE's and SAE's to the Protocol Chair, and all applicable parties.
- Facilitating audits by securing selected source documents and research records from participating sites for audit, or by auditing at participating sites.

Participating Sites

Participating sites are responsible for performing the following tasks:

- Following the protocol as written, and the guidelines of Good Clinical Practice (GCP).
- Submitting data to the Coordinating Center.
- Registering all patients with the Coordinating Center by submitting patient registration form, and signed informed consent promptly.
- Providing sufficient experienced clinical and administrative staff and adequate facilities and equipment to conduct a collaborative trial according to the protocol.
- Maintaining regulatory binders on site and providing copies of all required documents to the Coordinating Center.
- Collecting and submitting data according to the schedule specified by the protocol.

5.2.7 Patient Registration

Prior to protocol enrollment and initiation of treatment, subjects must sign and date an IRB approved consent form. All patients must be registered centrally at the Sidney Kimmel Comprehensive Cancer Center.

To register a patient, sites must send the following documents to Tearra Miles and Sheila Linden at the Coordinating Center by email at (tlawre8@jhmi.edu and slinden2@jhmi.edu).

- Registration Form
- HIPAA authorization form (if separate from consent)
- Eligibility screening checklist
- Copy of all required screening tests.

The Coordinating Center will review the documents to confirm eligibility. To complete the registration process, the Coordinating Center will:

- assign a patient study number
- register the patient on the study with the Sidney Kimmel Comprehensive Cancer Center's Clinical Research Office
- E-mail the patient study number to the participating site.

5.2.8 Randomization

Subjects who are eligible to enroll in the study will be randomized in a 1:1 ratio to receive CC486 or observation. Subjects will be randomized through a computer program maintained by the study team.

- CC-486 (three 100 mg tablets on Days 1-21 every 28 days)
- Observation

The randomization will be balanced by using randomly permuted blocks and will be stratified by the following criteria:

- Positive lymph nodes at time of resection (N0 versus N1)

Approximately 55-75% of patients are expected to be node positive.[10, 13, 40-42]

CC-486 will be packaged and designated for a patient upon randomization to Arm A. Subsequent bottles of study drug will be made available to patients as needed. Patient will receive one bottle of study drug with no more than 63 tablets in the bottle.

External sites will submit a patient enrollment log to the Coordinating Center monthly by email, at vrami2@jhu.edu and tlawre18@jhmi.edu.

5.2.9 Dosage Dispensing and Administration

Treatment will be administered on an outpatient basis. Reported adverse events and potential risks are described in Section 6. Appropriate dose modifications are described in Section

No investigational or commercial agents or therapies other than those described below may be administered with the intent to treat the patient's malignancy.

5.2.9.1 Dispensing and Administration of CC-486

On Day 1 of each cycle, subjects in Arm A will be administered three tablets of 100 mg of CC486 in clinic with approximately 8 ounces (240mL) of room temperature water. CC-486 may be taken on an empty stomach or with food. Dosing can occur at any time during the morning after completing the required pre-dose assessments. Time of dose should be recorded in study documents. Anti-emetics can be administered per discretion of the investigator (per Section 5.4.1) on an as needed basis.

For each cycle, subjects in Arm A will take the three 100 mg tablets of CC-486 on days 1-21 of a 28 day cycle. On the first day of every cycle, subjects will be given a quantity of study drug for the dosing days at home. Subjects should be instructed to inspect each CC-486 tablet and only take tablets that are totally intact. Subjects should be instructed to return any doses not taken or

any tablet(s) found to be not totally intact to the study clinic on the first day of each cycle, beginning with Cycle 2. Subjects should be instructed to open the study medication packaging as close as possible to when they are going to take the study drug and record the time of study drug administration on a diary card (Appendix B). The diary card will be returned to clinic staff at the end of each cycle. On days when study drug is taken at home, subjects should be encouraged to ingest CC-486 with approximately 8 ounces (240mL) of room temperature water. CC-486 may be taken on an empty stomach or with food.

If a dose of CC-486 is missed, it should be taken as soon as possible on the same day. If it is missed for the entire day, it should not be made up. If the patient vomits after a dose is taken, no more subsequent tablets should be administered.

The cycle length is 4 weeks (28 days). Treatment with protocol therapy will continue until unacceptable toxicities or subject has visible disease progression on imaging. Further details per Sections 5 and 7.

If CC-486 is shipped directly to patients for the safety of patients and staff as part of COVID-19 precautions, a courier that monitors drug shipment conditions should be utilized. Study staff must call the patient to ensure that shipment was received in good condition at the patient's home, and instruct patient to keep and return all empty, partially empty, or full bottles/blisters to the site at next visit for drug reconciliation. This call, including verbal confirmation of receipt by the patient, should be properly documented in the medical record. If COVID-19 precautions prevent the return of used/unused study drug supplies to the site, then study staff must review the remaining pills during a telemedicine visit. Undelivered supplies should be returned to the clinical site.

5.2.10 Record of administration

Accurate records will be kept in the source documents of all drug administration (including prescribing and dosing). Subject diaries will be collected at the end of each cycle and at the discontinuation of treatment.

Review of remaining pills and pill diary may be reviewed during a telemedicine visit when drug is unable to be returned to study staff due to COVID-19 precautions. Copies of the pill diary may be submitted to study staff by phone, fax, email, or mychart.

5.3 Visit Schedule, Assessments, and Dose Modifications

Screening assessments and all on-study scheduled visits and assessments are outlined in Section 2 Table of Study Assessments and detailed below.

5.3.1 Screening and Pre-Study Assessments

Screening for enrollment in this clinical trial should include a record of prior medications and anti-cancer treatments, a record of concomitant medications and procedures, medical history, a physical examination, vital signs, weight, ECOG performance status (per Appendix A), pregnancy test, labs (CBC with differential, serum chemistry and hepatic panel, CA 19-9, PT, PTT), research labs, quality of life measures and lesion assessment with CT (per Section 7). All pre-study assessments should be done ≤ 28 days from Cycle 1 Day 1.

5.3.2 Initial Phase: Assessments, Dose Modifications, and Termination

5.3.2.1 Arm A for first cycle

Prior to receiving Cycle 1 Day 1 of CC-486, patients must undergo a physical examination including vital signs, weight, and ECOG performance status assessments, and labs including CBC with differential, serum chemistry and hepatic panel, CA 19-9, PT/PTT, and research blood, a record of concomitant medications and procedures, quality of life, and an assessment of adverse events. If screening assessments were done within 7 days of initiating Cycle 1 Day 1, these do not need to be repeated. Cycle 1 of CC-486 in Arm A can then be administered on schedule if treatment criteria are met.

5.3.2.2 Arm A for first 12 cycles

Arm A patients will be evaluated on Day 1 of every cycle with a physical examination, ECOG performance status, vital signs, weight, CBC with differential, research blood and serum chemistry and hepatic panel. Arm A patients will also complete an assessment of adverse events and reporting of current medications/procedures on Day 1 of every cycle.

Arm A patients will complete a CA 19-9, quality of life assessment and CT scan on Day 1 of every third cycle beginning at Cycle 4.

Arm A subjects will be evaluated for adverse events on Day 1 of each cycle with the NCI CTCAE v4.0 (**http://ctep.info.nih.gov**) used as a guide for the grading of severity. For Arm A patients, doses will be reduced for hematologic and other non-hematologic toxicities that can be attributed to the study drug. Dose adjustments are to be made according to the system showing the greatest degree of toxicity. When a dose reduction is required for Day 1 of any cycle, no dose re-escalation will be permitted for the duration of study treatment.

NOTE: Arm B patients will not be evaluated for adverse events as they do not receive the study agent.

Criteria to Initiate Treatment

In Arm A, a new course of treatment may begin on the scheduled Day 1 of a new cycle if:

- Absolute neutrophil count ≥ 1500/mm³
- Platelet count ≥ 100,000/mm³
- Serum creatinine levels < 2 mg/dL
- Serum bilirubin levels ≤ 1.5 mg/dL.
- AST (SGOT) and ALT (SGPT) ≤ 2.5 x the institutional upper limit (ULN) of the normal range
- Any other drug-related adverse events that may have occurred must have resolved to < Grade 2 severity except grade 2 anorexia and any correctable metabolic abnormality.
- No evidence of visible disease recurrence on imaging (if indicated per Study Schedule, Section 2, to be done prior to cycles 4, 7, and 10)

If these conditions are not met on Day 1 of a new cycle, the subject will be evaluated weekly and a new cycle of treatment will not be initiated until the toxicity has resolved as described above utilizing the dose modifications as depicted below. If the study drug has to be held for > 28 days, study treatment will be discontinued.

5.3.2.3 Arm B for the first 12 cycles

Arm B patients will ideally be evaluated (or at the very least contacted by the study team) on Day 1 of every third cycle beginning with Cycle 4.

Patients are permitted to see their local oncologist, and CA19-9 and lesion assessments may be completed outside of the recommended 3 month window (Cycles 4, 7, and 10) at their oncologist's discretion. .

Arm B patients will follow a similar schedule as patients in Follow-up, except for a Quality of Life assessment that will be performed.

Correlative blood draw is optional for Arm B patients. If Arm B patients wish, they may have research bloods drawn for correlative studies.

Arm B patients who opt to have research blood collected will have it collected on Day 1 of every third cycle (cycle 4, 7 and 10).

Below is the list of information that must be obtained from Arm B patients.

- Any further therapy received (records to be obtained)
- Subjective response on therapy (records to be obtained)
- Any CA 19-9 drawn (records to be obtained)
- Any imaging (images to be obtained)

Arm B patients will complete a CA 19-9, quality of life assessment and CT scan (or alternate imaging, if clinically indicated) quarterly (ideally).

Arm B patients who are not evaluated in person may have the Quality of Life assessment completed over the phone.

5.3.2.4 Dose Modification

Table 5.3.1: Arm A: Dose Modification Recommendations for CC-486 Phase

Toxicity	Grade	CC-486
**Febrile Neutropenia**	Grade 3 or 4	Hold until resolved and neutrophils ≥ 1500/mm^3<br>Restart study drug at 200 mg (two tablets, 33% dose reduction).<br>If the same Grade 3 or 4 event recurs (i.e., third occurrence) despite CC-486 dose reduction to 200 mg, as described above, permanently discontinue study
**Leukopenia and/or Neutropenia**	Grade 2-3	Hold dose until recovery to Grade ≤ 1 or baseline.<br>Resume study drug at prior dose
	Grade 4	Hold until resolved to ≤ Grade 1<br>Restart study drug at 200 mg (two tablets, 33% dose reduction).<br>If the same Grade 4 event recurs (i.e., third occurrence) despite CC-486 dose reduction to 200 mg, as described above, permanently discontinue study drug.
**Thrombocytopenia**	Grade 1* or 2	Hold treatment until resolution to normal values.
	Grade 3 or 4	Hold until resolved and platelet count ≥100,000/mm^3<br>Restart study drug at 200 mg (two tablets, 33% dose reduction)..
**Diarrhea** (unresponsive to maximum medical management)	Grade 1 or 2	Consider administering symptomatic remedies and/or start prophylaxis.<br>No dose/schedule changes

Toxicity	Grade	CC-486
	Grade 3 or 4	Hold until resolved to ≤ Grade 1<br>Restart study drug at 200 mg (two tablets, 33% dose reduction).
**Nausea/Vomiting** (unresponsive to maximum medical management)	Grade 1 or 2	Consider changing/adding antiemetic medication treatment<br>No dose/schedule changes
	Grade 3 or 4	Hold until resolved and neutrophils ≥ 1000/mm3<br>Restart study drug at 200 mg (two tablets, 33% dose reduction).

*Grade 1 thrombocytopenia: Platelet count < 100,000/mm^3

Study drug should be stopped if grade 3 or 4 toxicity has occurred despite a dose reduction to 66% of original dose (200 mg of CC-486 in two 100 mg tablets).

If grade 3 or 4 toxicities require patients to stop treatment mid cycle, they can resume their cycle at the recommended decreased dose if the toxicities resolve to ≤ Grade 1 within their 21 days of treatment (i.e. they would only continue treatment to complete their days 1-21 per the calendar, not to complete 21 days of taking the tablets). If their toxicities recover after their 21 days has completed, they will not take more tablets until they begin their next cycle.

5.3.2.5 Arm A: Missing doses

Missed Doses Day 1 dose
As Day 1 dose of CC-486 will be given in the office, this dose should not be missed. If the patient does not meet the criteria to begin a new cycle, then the patient will be reassessed in a week and Cycle X Day 1 will start when the dose is actually given.
Day X dose missed
If the dose held or missed was to be given on any day other than Day 1, the cycle continues per protocol with one dose not given and that dose is just considered omitted.

5.4 Discontinuation of Treatment: Arm A

In the absence of treatment delays due to adverse events, treatment with CC-486 may continue until one of the following criteria applies:

- Interruption of scheduled therapy for greater than four weeks,

- Intercurrent illness that prevents further administration of treatment,
- Adverse event(s) that, in the judgment of the Investigator, may cause severe or permanent harm or which rule out continuation of the treatment regimen
- For any grade 3 and 4 toxicity that recurs despite dose reduction
- Discontinuation of CC-486 for any reason,
- Treatment with other chemotherapeutic or investigational anti-neoplastic drugs,
- Subject decides to withdraw from the study,
- Suspected pregnancy or positive pregnancy, or
- General or specific changes in the subject's condition render the subject unacceptable for further treatment in the judgment of the investigator, or
- Disease recurrence on imaging, or
- Patient has completed one year of therapy with CC-486.

If the subject has visible disease recurrence on imaging or meets any of the aforementioned criteria for removal from study treatment, then the CC-486 or observation phase of this study will conclude and patient will enter the follow-up phase of the study, following the schedule of assessments as described in Section 5.4.2.9.

Mandatory research bloods and tumor biopsies will be obtained at the time of recurrence for both Arm A and Arm B subjects, per the method determined best per the investigator as outlined on the Study Calendar and in Section.

5.4.1 End of Treatment

When coming off of CC-486 therapy, subjects in Arm A will undergo a physical examination, vital signs, weight, ECOG performance status, assessment of adverse events, labs including CBC with differential, serum chemistry and hepatic panel, CA 19-9, PT/PTT and research blood. If any of these were performed within 7 days, they do not need to be repeated. All remaining pills will be collected.

5.5 Follow-up Phase Both Arms

<u>No disease recurrence:</u>
Less than 10% of patients who have undergone surgery will not develop tumor recurrence after adjuvant therapy.
Therefore, if patients have not recurred after one year of therapy with CC- 486 (Arm A) the drug will be discontinued.

Patients in Arm A will undergo a safety assessment 28 days (± 2 days) post the last dose of protocol therapy for resolution of toxicity.
Because Arm B patients do not receive study drug, a safety assessment will not be performed.

All patients (Arm A and B) will be then be contacted (ideally) every 3 months on to obtain the following information about recurrence, survival and anti-cancer treatment (2.2 **Study Calendar**):

- Any further therapy received (records to be obtained)
- Subjective response on therapy (records to be obtained)
- Any CA 19-9 drawn (records to be obtained)
- Any imaging (images to be obtained)

Disease recurrence
At the time of recurrence, Arm A and Arm B subjects will undergo biopsy of the recurrent lesion per the method determined best per the investigator.
Post recurrence images will be also obtained (pre-recurrence images are optional)
Thereafter, attempts to contact patients every 3 months in order to obtain lesion assessments/post recurrence images, CA19-9 and anticancer treatment and survival information as detailed in section 5.4.2. If study team is unable to make contact with patients, information may be obtained through medical and public records. This information will continue to be collected for patients until patients hit second disease progression.

5.6 Concomitant Therapy

5.6.1 Recommended Concomitant Therapy

Subjects should receive full supportive care, including transfusions of blood and blood products, antibiotics, antiemetics, and growth factor support when appropriate.

1. It is recommended that all subjects must be pre-medicated for nausea and vomiting prior to the administration of CC-486. Administration of a 5-HT3 serotonin receptor antagonist is recommended prior to CC-486. Antiemetics should be administered at least 30 minutes prior to administration of CC-486. Additional doses may be administered if required. Other antiemetic medications (prochlorperazine, lorazepam, etc.) may also be taken for delayed nausea as needed. Specific anti-emetic regimens are up to the discretion of the investigator.

2. Diarrhea: If diarrhea occurs during dosing, an antidiarrheal agent(s) may be used. It is recommended that patients experiencing diarrhea associated with CC-486 be managed according to the guidelines provided in Appendix C, which are based on a publication in

the Journal of Clinical Oncology[43]. A dose reduction of CC-486 may be appropriate based on the severity of diarrhea observed and the response to treatment intervention.

3. Contraceptive Therapy: Sexually active men and women of child-bearing potential must agree to use effective contraception.

5.6.2 Prohibited Concomitant Therapy

1. Concomitant use of other anti-cancer therapies, including radiation, or other investigational agents is not permitted while subjects are receiving protocol therapy during the treatment phase of the study.

5.7 Treatment adherence

Research center personnel will review the dosing instructions with subjects. Subjects will be asked to maintain a diary to record CC-486 administration (see Appendix B). Subjects will be asked to bring any unused drug and empty drug containers to the research center at their next visit. Research personnel will count and record the number of used and unused drug at each visit and reconcile with the medication diary.

6 Adverse events

6.1 Definitions Adverse Event (AE) Definition

An adverse event is the development of an undesirable medical condition or the deterioration of a pre-existing medical condition following or during exposure to a pharmaceutical product, whether or not considered causally related to the product. An undesirable medical condition can be symptoms (e.g., nausea, chest pain), signs (e.g., tachycardia, enlarged liver) or the abnormal results of an investigation (e.g., laboratory findings, electrocardiogram). In clinical trials, from the time of signing an informed consent, an AE can include an undesirable medical condition occurring at any time, including run-in or washout periods, even if no trial treatment has been administered. For this trial, Adverse Events and Serious Adverse Events will be collected for Arm A patients only, as they are receiving study drug.

6.1.1 Serious Adverse Event (SAE) Definition

A serious adverse event is one that at any dose (including overdose):

- Results in death
- Is life-threatening[1]
- Requires inpatient hospitalization or prolongation of existing hospitalization
- Results in persistent or significant disability or incapacity[2]
- Is a congenital anomaly or birth defect

- Is an important medical event[3]
- Pregnancy

1. “Life-threatening” means that the subject was at immediate risk of death at the time of the serious adverse event; it does not refer to a serious adverse event that hypothetically might have caused death if it were more severe.
2. “Persistent or significant disability or incapacity” means that there is a substantial disruption of a person’s ability to carry out normal life functions.
3. Medical and scientific judgment should be exercised in deciding whether expedited reporting is appropriate in situations where none of the outcomes listed above occurred. Important medical events that may not be immediately life-threatening or result in death or hospitalization but may jeopardize the patient or may require intervention to prevent one of the other outcomes listed in the definition above should also usually be considered serious. Examples of such events include allergic bronchospasm requiring intensive treatment in an emergency room or at home, blood dyscrasias or convulsions that do not result in inpatient hospitalization, or the development of drug dependency or drug abuse. A new diagnosis of cancer during the course of a treatment should be considered as medically important.

6.2 Adverse Event Reporting

Protocol Chair

The Protocol Chair is ultimately responsible for the required reporting of all adverse events.

Coordinating Center

The Coordinating Center is the central location for the collection and maintenance of documentation of adverse events and is responsible for submitting adverse event reports to the Protocol Chair promptly. The Coordinating Center will maintain documentation of all adverse event reports for each participating site. Adverse event reports submitted to the Coordinating Center must be signed and dated by the participating site’s Principal Investigator. Participating sites must use the SKCCC template for reporting adverse events per SKCCC policy. Information to be provided must include:

- Subject ID number, and initials
- Date of the event
- Description of the event
- Description of site's response to the event
- Assessment of the subject's condition
- Subject's status on the study (on study, off study, etc.)
- Attribution of event to study drug

Participating Sites

Participating sites are responsible for reporting adverse events to their IRB according to its specific requirements and to the Coordinating Center as follows:

Fatal Events whether anticipated or unanticipated, and whether or not related to the study must be reported to the Coordinating Center within **24 hours** of the participating site Principal Investigator's learning of the event.

Serious and Unanticipated Adverse Events as defined above must be reported to the Coordinating Center within **24 hours** of the participating site Principal Investigator's learning of the event.

Other Serious Adverse Events which may result in a change to the protocol, informed consent, or risk to subjects as specified in the protocol must be reported within **three (3) working days** of the participating site Principal Investigator's learning of the event.

Adverse Events which result in no change to protocol, informed consent, or risk to subjects must be reported to the Coordinating Center on a monthly basis.

Adverse event reports are to be emailed to the Coordinating Center at GISafetyReporting@jhmi.edu and tlawre18@jhmi.edu. Follow-up reports are to be emailed to the Coordinating Center as necessary.

The investigator must also report follow-up information about SAEs within the same time frames.

If a non-serious AE becomes serious, this and other relevant follow-up information must also be provided within the same time frames described above.

All SAEs for Arm A patients must be collected whether or not they are considered causally related to the investigational product. Investigators and other site personnel are responsible for reporting all casually related SAEs to their IRB and the Protocol Chair.

All SAE's should be reported to the Coordinating Center by email at GISafetyReporting@jhmi.edu and cc the Study Coordinator, Tearra Miles at tlawre18@jhmi.edu.

Toxicity will be scored using CTCAE Version 4.0 for toxicity and adverse event reporting. A copy of the CTCAE Version 4.0 can be downloaded from the CTEP homepage

(**http://ctep.info.nih.gov**). All appropriate treatment areas should have access to a copy of the CTCAE Version 4.0. All adverse clinical experiences, whether observed by the investigator or reported by the subject, must be recorded, with details about the duration and intensity of each episode, the action taken with respect to the test drug, and the subject's outcome. The investigator must evaluate each adverse experience for its relationship to the test drug and for its seriousness. The investigator must appraise all abnormal laboratory results for their clinical significance. If any abnormal laboratory result is considered clinically significant, the investigator must provide details about the action taken with respect to the test drug and about the subject's outcome. Only clinically significant lab abnormalities will be recorded as adverse events.

6.2.1 Pregnancies

Pregnancy of a female subject or the female partner of a male subject occurring while the subject is on CC-486, or within 4 weeks after the subject's last dose of CC-486, are considered expedited reportable events. If the subject is on CC-486, it is to be discontinued immediately. The pregnancy must be reported to Celgene Drug Safety within 24 hours of the Investigator's knowledge of the pregnancy by phone and facsimile using the SAE Form. The female subject may be referred to an obstetrician-gynecologist (not necessarily one with reproductive toxicity experience) or another appropriate healthcare professional for further evaluation.

The Investigator will follow the pregnant female until completion of the pregnancy, and must notify Celgene Drug Safety of the outcome of the pregnancy (either normal or abnormal outcome) as specified below. The Investigator will provide this information as a follow-up to the initial SAE.

If the outcome of the pregnancy meets the criteria for immediate classification as a SAE (i.e., spontaneous abortion [any congenital anomaly detected in an aborted fetus is to be documented], stillbirth, neonatal death, or congenital anomaly), the Investigator should follow the procedures for Expedited Reporting of SAEs to Celgene (i.e., report the event to Celgene Drug Safety by facsimile within 24 hours of the Investigator's knowledge of the event).

All neonatal deaths that occur within 30 days of birth should be reported, without regard to causality, as SAEs. In addition, any infant death after 30 days that the Investigator suspects is related to the *in utero* exposure to CC-486 should also be reported. In the case of a live "normal" birth, Celgene Drug Safety should be advised as soon as the information is available.

If a female partner of a male subject taking CC-486 becomes pregnant, the male subject taking the project should notify the Investigator, and the pregnant partner should be advices to call their healthcare provider immediately.

6.2.2 Celgene Drug Safety Contact Information:

Celgene Corporation
Global Drug Safety and Risk Management
Connell Corporate Park 300 Connell Dr.
Suite 6000
Berkeley Heights, NJ 07922
Fax: (908) 673-9115
E-mail: drugsafety@celgene.com

6.3 Investigator Reporting Responsibilities

The conduct of the study will comply with all FDA safety reporting requirements.

6.3.1 Expedited reporting by investigator to Celgene

Serious adverse events (SAE) are defined above. The investigator must inform Celgene in writing using a Celgene SAE form or MEDWATCH 3500A form of any SAE within 24 hours of being aware of the event. The written report must be completed and supplied to Celgene by facsimile within 24 hours/1 business day. The initial report must be as complete as possible, including an assessment of the causal relationship between the event and the investigational product(s), if available. Information not available at the time of the initial report (e.g., an end date for the adverse event or laboratory values received after the report) must be documented on a follow-up report. A final report to document resolution of the SAE is required. The Celgene tracking number (**OA-PANC-PI-0294**) and the institutional protocol number should be included on SAE reports (or on the fax cover letter) sent to Celgene. A copy of the fax transmission confirmation of the SAE report to Celgene should be attached to the SAE and retained with the subject records.

6.3.2 Report of Adverse Events to the Institutional Review Board

The principal Investigator is required to notify his/her Institutional Review Board (IRB) of a serious adverse event according to institutional policy.

6.3.3 Investigator Reporting to the FDA

Serious adverse events (SAEs) that are **unlisted/unexpected, and at least possibly associated to 5-azacitadine,** and that have not previously been reported in the Investigator's brochure, or reference safety information document should be reported promptly to the Food and Drug Administration (FDA) by telephone or by fax by the IND sponsor. Fatal or life threatening SAEs that meet the criteria for reporting to the FDA must be reported to the FDA within 7 calendar days after awareness of the event. All other SAEs that meet the criteria for reporting to the FDA must be reported to the FDA within 15 calendar days after awareness of the event by the IND sponsor.

A clear description of the suspected reaction should be provided along with an assessment as to whether the event is drug or disease related.

In accordance with the regulation 21 CFR § 312.33, the IND Sponsor shall within 60 days of the anniversary date that the IND went into effect submit a brief report of the adverse events and progress of the investigation. Please refer to Code of Federal Regulations, 21 CFR § 312.33 for a list of the elements required for the annual report. All IND annual reports will be submitted to the FDA by the IND Sponsor.

6.4 Adverse event updates/IND safety reports

Celgene shall notify the Investigator via an IND Safety Report of the following information:

- Any AE associated with the use of drug in this study or in other studies that is both serious and unexpected.
- Any finding from tests in laboratory animals that suggests a significant risk for human subjects including reports of mutagenicity, teratogenicity, or carcinogenicity.

The Investigator shall notify his/her IRB/EC promptly of these new serious and unexpected AE(s) or significant risks to subjects.

The Investigator must keep copies of all AE information, including correspondence with Celgene and the IRB/EC, on file (see Section 12.4 for records retention information).

7 Radiologic Evaluation

7.1 Baseline imaging assessments

Baseline imaging assessment must occur within ≤28 days of initiation of the CC-486 /observation phase to document lack of visible disease on imaging as indicated in Section 2, Schedule of Study Assessments. Acceptable imaging modalities are described below and must include imaging of the chest, abdomen and pelvis. To be declared free of visible disease, there can be no evidence of measurable disease, as defined below, or presence of malignant effusions.

All effusions that can safely be diagnostically drained must be evaluated prior to enrolling to document lack of malignant cells via thoracentesis or paracentesis. Fluid that is non-specific or too small to drain is considered no visible disease. If there is a concern for bone metastasis or brain metastasis based on clinical symptoms, bone scan and/or brain MRI must be done to rule out metastatic disease prior to enrolling on this study. These scans do not need to be done in the absence of concerning clinical symptoms.

Imaging will be performed every 3 cycles while on the CC-486. Ideally, imaging will be performed every 3 cycles for patients on the observation arm, but ultimately imaging/lesion assessments will be performed at the patient or his/her oncologist's discretion. When patients have evidence of measurable disease, as defined below, they will then be classified as having "visible disease". If a lesion or effusion appears but is not confirmed to be disease until a later time point, the time noted for a patient to have "visible disease" should revert back to the time the lesion or effusion initially appeared. They will stop the CC-486 /observation phase and enter the follow up phase. Imaging assessment ideally should occur within ≤ 28 days of initiation of first-line chemotherapy. If there is a delay between initial documentation of visible disease and starting treatment, imaging may be repeated as indicated in Section 2, Schedule of Study Assessments to get a true baseline.

7.2 Definitions of Measurable and Non-Measurable Disease

- Measurable disease is defined as at least one lesion whose longest diameter can be accurately measured as > 1.0 cm with CT scan, CT component of a PET/CT, or MRI. Clinical lesions will only be considered measurable if they meet the conditions of Section 7.3.

- Note: Tumor lesions that are situated in a previously irradiated area might or might not be considered measurable.

- Malignant lymph nodes. To be considered pathologically enlarged and measurable, a lymph node must be > 1.5 cm in short axis when assessed by CT scan (CT scan slice thickness recommended to be no greater than 5 mm). At baseline and in follow-up, only the short axis will be measured and followed.

- All other lesions (or sites of disease), including small lesions (longest diameter < 1 cm or pathological lymph nodes with ≥ 1 to <1.5 cm short axis) are considered nonmeasurable disease. Bone lesions, leptomeningeal disease, ascites, pleural/pericardial effusions, lymphangitis cutis/pulmonis, inflammatory breast disease, and abdominal masses (not followed by CT or MRI), are considered non-measurable.

- Note: Cystic lesions that meet the criteria for radiographically defined simple cysts should not be considered as malignant lesions (neither measurable nor non-measurable) since they are, by definition, simple cysts. 'Cystic lesions' thought to represent cystic metastases can be considered as measurable lesions, if they meet the definition of measurability described above. However, if non-cystic lesions are present in the same subject, these are preferred for selection as target lesions.

7.3 Guidelines for Evaluation of Measurable Disease

7.3.1 Measurement Methods

- All measurements should be recorded in metric notation (i.e., decimal fractions of centimeters) using a ruler or calipers.
- The same method of assessment and the same technique must be used to characterize each identified and reported lesion at baseline and during follow-up.
- Imaging-based evaluation is preferred to evaluation by clinical examination when both methods have been used at the same evaluation to assess the antitumor effect of a treatment.
- All measurements will be made based on RECIST guideline version 1.1[44]

7.3.2 Acceptable Imaging Modalities for Measurable Disease

- CT chest, abdomen and pelvis with IV contrast is the preferred modality to assess the presence of visible disease in study patients and then to follow their response to chemotherapy. When CT cannot be used, MRI chest, abdomen and pelvis is acceptable. If there is a lesion that is uncertain as to whether it is disease or not, PET-CT can be used to make this determination per the investigator's discretion.
- Conventional CT: This guideline has defined measurability of lesions on CT scan based on the assumption that CT slice thickness is 5 mm or less. If CT scans have slice thickness greater than 5 mm, the minimum size for a measurable lesion should be twice the slice thickness. CT scans should be the default mode of assessing for visible disease in all patients.
- Clinical Lesions: Clinical lesions will only be considered measurable when they are superficial (e.g., skin nodules and palpable lymph nodes) and >10 mm diameter as assessed using calipers (e.g., skin nodules). In the case of skin lesions, documentation by color photography, including a ruler to estimate the size of the lesion, is recommended.
- Ultrasound (US): Ultrasound is not useful in assessment of lesion size and should not be used as a method of measurement. Ultrasound examinations cannot be reproduced in their entirety for independent review at a later date and, because they are operator dependent, it cannot be guaranteed that the same technique and measurements will be taken from one assessment to the next. If new lesions are identified by ultrasound in the course of the study, confirmation by CT or MRI is advised. If there is concern about radiation exposure at CT, MRI may be used instead of CT in selected instances.

7.3.4 Measurement of Effect

Response and progression will be evaluated in this study using the new international criteria proposed by the revised Response Evaluation Criteria in Solid Tumors (RECIST) guidelines (version 1.1)[44]. Radiologists are our institution will be interpreting the films and the principle investigator will be determining the type of response or progression using the RECIST criteria.

7.3.4.1 Target Lesions

- All measurable lesions (as defined in Section 7.2) up to a maximum of 2 lesions per organ and 5 lesions in total, representative of all involved organs, should be identified as target lesions and recorded and measured at baseline. If the protocol specified studies are performed, and there are fewer than 5 lesions identified (as there often will be), there is no reason to perform additional studies beyond those specified in the protocol to discover new lesions.

- Target lesions should be selected on the basis of their size (lesions with the longest diameter), be representative of all involved organs, but in addition should be those that lend themselves to reproducible repeated measurements. It may be the case that, on occasion, the largest lesion does not lend itself to reproducible measurement in which circumstance the next largest lesion which can be measured reproducibly should be selected.

- Baseline sum of Diameters (BSD): A sum of the diameters (longest for non-nodal lesions, short axis for nodal lesions) for all target lesions will be calculated and reported as the baseline sum of diameters. If lymph nodes are to be included in the sum, then only the short axis is added into the sum. The BSD will be used as reference to further characterize any objective tumor response in the measurable dimension of the disease.

7.3.4.2 Non-Target Lesions

All other lesions (or sites of disease) should be identified as non-target lesions and should also be recorded at baseline. Measurements are not required, and these lesions should be followed in accord with Section 7.3.5.2.

7.3.5 Response Criteria during Follow-up Phase

All identified sites of disease must be followed on reevaluation. Specifically, a change in objective status to either a PR or CR cannot be done without rechecking all identified sites (i.e., target and non-target lesions) of pre-existing disease.

7.3.5.1 Evaluation of Target Lesions

- Complete Response (CR): Disappearance of all target lesions *and normalization of tumor biomarkers (CA 19-9).* Any pathological lymph nodes (whether target or non-target) must have reduction in short axis to < 1 cm.

- Partial Response (PR): At least a 30% decrease in the sum of the diameters of target lesions taking as reference the BSD.

- Progression (PD): At least a 20% increase in the sum of diameters of target lesions taking as reference the smallest sum of diameters recorded on study (this includes the baseline sum if that is the smallest on study) or the appearance of one or more new lesions. In addition to the relative increase of 20%, the sum must also demonstrate an absolute increase of at least 5 mm.

- Stable Disease (SD): Neither sufficient shrinkage to qualify for PR nor sufficient increase to qualify for PD taking as references the smallest sum of diameters while on study.

7.3.5.2 Evaluation of Non-Target Lesions

- Complete Response (CR): Disappearance of all non-target lesions and normalization of tumor biomarker level. All lymph nodes must be non-pathological in size (< 1 cm short axis)

- Non-CR/Non-PD: Persistence of one or more non-target lesions and/or maintenance of tumor biomarker level above the normal limits.

- Progression (PD): Appearance of one or more new lesions. Unequivocal progression of existing non-target lesions. Unequivocal progression should not normally trump target lesion status. It must be representative of overall disease status change, not a single lesion increase.

- NOTE: Although a clear progression of "non-target" lesions only is exceptional, in such circumstances, the opinion of the treating physician will prevail, and the progression status will be confirmed at a later time by the study chair or a review panel.

7.3.6 Overall Objective Status during Follow-up Phase

The overall objective status for an evaluation is determined by combining the subject's status on target lesions, non-target lesions, and new disease as defined in the following tables (Table 7.3.6.1, 7.3.6.2)

Table 7.3.6.1 For Subjects with Measurable Disease (i.e., Target Disease):

Target Lesions	Non-Target Lesions*	New Lesions	CA 19-9	Overall Objective Status
CR	CR	No	Normal (≤ ULN)	CR
CR	Non-CR/Non-PD/not evaluated	No	Normal	PR
PR	Non-CR/Non-PD/not evaluated	No	Any value	PR
SD	Non-CR/Non-PD/not evaluated	No	Any value	SD
PD	Any	Yes or No	Any value	PD
Any	PD	Yes or No	Any value	PD
Any	Any	Yes	Any value	PD
	* ‘Non-CR/non-PD’ is preferred over ‘stable disease’ for non-target disease since SD is increasingly used as an endpoint for assessment of efficacy in some trials so to assign this category when no lesions can be measured is not advised.			

Table 7.3.6.2 For Subjects with Non-Measurable Disease (i.e., Non-Target Disease):

Non-Target Lesions	New Lesions	CA 19-9	Overall Objective Status
CR	No	Normal	CR
Non-CR/non-PD	No	Any value	Non-CR/non-PD*
Not all evaluated	No	Any value	not evaluated
Unequivocal PD	Yes or No	Any value	PD
Any	Yes	Any value	PD
	* ‘Non-CR/non-PD’ is preferred over ‘stable disease’ for non-target disease since SD is increasingly used as an endpoint for assessment of efficacy in some trials so to assign this category when no lesions can be measured is not advised.		

7.3.6.1 Residual Disease

In some circumstances it may be difficult to distinguish residual disease from normal tissue. When the evaluation of complete response depends upon this determination, it is recommended that the residual lesion be investigated (fine needle aspirate/biopsy) before confirming the complete response status.

7.3.6.2 Symptomatic Deterioration

Subjects with global deterioration of health status requiring discontinuation of treatment without objective evidence of disease progression at that time, and not either related to study treatment or other medical conditions, should be reported as PD due to "symptomatic deterioration." Examples of symptomatic deterioration include:

- Weight loss >10% of body weight.
- Worsening of tumor-related symptoms.
- Decline in performance status of >1 level on ECOG scale.

Every effort should be made to document the objective progression even after discontinuation of treatment due to symptomatic deterioration.

8 Correlative Studies

Research samples will be collected at the discretion of the PI based on availability of supplies and safety of patient and staff.

Two sources of tumor DNA, blood and tumor biopsies will be evaluated for DNA methylation status in each subject to determine if therapy with CC-486 results in demethylation. These studies will also evaluate for an epigenetic signature that can help predict patients who will be responders versus non-responders and identify predictive signatures of possible recurrence and benefits of CC-486 therapy.

8.1 Plasma/Serum Samples

Blood for correlative studies will be drawn from all subjects in the study at screening and on Cycle 1 Day 1 prior to starting treatment. If screening assessments were done within 7 days of initiating Cycle 1 Day 1, these do not need to be repeated. Arm B patients have optional research blood draws on Day 1 Cycle 1 and on Day 1 of every third cycle (Cycle 4, 7, 10, etc.) thereafter. Blood for correlative studies will also be drawn from all patients at disease progression during the initial CC-486/observation phase. The blood samples will be used to assess the methylation status of free tumor DNA circulating in the blood. Both global and candidate gene methylation patterns

will be evaluated in plasma samples. Some of the potential candidate genes include: *TMS1, ARH1, hMLHI.*

Quantitative analysis using real-time Methylation-specific PCR (MSP) will be used to detect the presence of methylated alleles for specific genes. Genes found to be methylated in pretreatment samples will be monitored for changes during and after treatment using real-time MSP. DNA will first be extracted from plasma using standard phenol chloroform extraction. The DNA will then be analyzed for changes in DNA methylation at specific gene promoters that include *TMS1, ARH1, hMLHI*. PCR primers for these have been described and are in frequent use in the Azad/Yarchoan lab. This strategy of real-time MSP has recently shown promising results in an ongoing trial in metastatic lung cancer with combination epigenetic therapy with subcutaneous 5-azacitidine and the HDAC inhibitor, entinostat.

We will also do methylation status analysis on samples using Methylation-on-Beads (MOB), a novel nano-enabled methylation based technology. This assay has shown great promise as a tool for surveillance of responses to epigenetic therapy in preliminary studies in solid tumors. The utility of the nano-enabled platform will also be explored in the ongoing correlative studies since this may provide a more sensitive assay for detection of response to epigenetic therapy than real time MSP.

8.2 Tumor Biopsies

Tumor biopsies will be attempted on all subjects at the time of visible disease recurrence. We will also consent subjects to obtain paraffin-embedded blocks of their original resected pancreatic tumor. Methylation status will be assessed on these samples to correlate with the data received from the plasma analysis. All new biopsy specimens will be stored as fresh tissue at ≤ -70°C for extraction of DNA and RNA.

All biopsy specimens will undergo tumor methylation analysis with MSP for select candidate genes (*TMS1*, *ARH1*, etc) as described above. Evaluation of expression of genes that become demethylated post-treatment will be performed using quantitative real-time PCR, whenever possible. Where sufficient tissue is obtained, samples will also be studied using an Infinium Methylation Assay that surveys genome-wide DNA methylation profiles (such as the Illumina HumanMethylation27 BeadChip or the newer Infinium HumanMethylation450 BeadChip). This technology examines approximately 27,500 CpGs in 14,500 regions, including 12,833 well-annotated genes described in the NCBI CCDS database, 144 methylation hotspots in cancer genes, 982 cancer related targets, and 110 microRNA promoters. Results are analyzed using BeadStudio software that allows for data analysis and integration of methylome data with mRNA or miRNA expression array studies. In regions of cancer hotspots, there are 7 CpGs studied on average per

gene. As little as 500ng of input DNA is required for the BeadChip, which allows for investigation of samples collected by laser capture microdissection. We will investigate if the Illumina Infinium chip can be used to define epigenetic signatures between responders and non-responders to oral epigenetic therapy.

8.2.1 Specimen Collection and Handling

8.2.1.1 Blood

Blood samples for methylation analysis will be collected from all participants in EDTA BD Vacutainer cell preparation tubes (purple tubes, Becton Dickinson, Franklin Lakes, NJ). These tubes are designed to separate plasma and mononuclear cells from whole blood. Forty to sixty mL of blood will be collected from each subject during each collection as per Section 2, Schedule of Study Assessment. For samples drawn at Johns Hopkins, tubes will be transported on ice and processed within 30 minutes in Dr. Azad/ Yarchoan's laboratory. Blood collected at other sites will follow the process for shipping samples to Johns Hopkins Hospital as described in the Laboratory Manual for the study. All blood not used for correlative analyses will be stored for future use if new biomarkers are developed that may better predict recurrence.

8.2.1.2 Archival Tissue

Patients will be consented in order for us to obtain paraffin-embedded blocks and slides of their original resected tumor. This will enable us to perform methylation analysis on the primary tumor and compare this to the tumors at the time of recurrence to determine if treatment with CC-486 led to any changes in methylation. The first slide should be stained for H&E (Hematoxylin and Eosin) for confirmation of the presence of tumor tissue; then, up to 20 serially cut slides should follow. Slides should be 10 microns in thickness and placed in tubes; alternatively, sections may be mounted on charged or silanted slides without coverslips. The H&E stained and all unstained slides will be submitted.

8.2.1.3 Tumor Biopsies

At the time of recurrence, there must be an attempt to obtain tumor samples by FNA or core biopsy from accessible tumor tissue. A maximum of 8 core biopsies (or fine needle aspirates if cores are considered unsafe) will be obtained from each subject. The aspirates will be used to prepare air dried cytologic smears and frozen samples. The first pass will be used for on-site examination of the material, to determine presence and quality of lesional tissue. The remaining biopsy specimens will be flash frozen in liquid nitrogen and then stored as fresh tissue at ≤ -70°C for extraction of DNA and RNA. The biopsy specimens will be stored and evaluated in Dr. Azad/ Yarchoan's laboratory.

8.2.1.4 Subject Anonymity

A unique subject identifier will be assigned to each subject and will be used to identify tumor biopsies and blood samples. The protocol scientific investigator(s) handling the samples will be blinded as to the subject identification, subject data and outcome.

9 Protocol Amendments/Deviations

9.1 Protocol Amendments

Any amendment to this protocol must be agreed to by the Principal Investigator and reviewed by Celgene. Amendments should only be submitted to IRB/EC after consideration of Celgene review. Written verification of IRB/EC approval will be obtained before any amendment is implemented.

9.2 Protocol Deviations

When an emergency occurs that requires a deviation from the protocol for a subject, a deviation will be made only for that subject. A decision will be made as soon as possible to determine whether or not the subject (for whom the deviation from protocol was effected) is to continue in the study. The subject's medical records will completely describe the deviation from the protocol and state the reasons for such deviation. In addition, the Investigator will notify the IRB/EC in writing of such deviation from protocol. Non-emergency minor deviations from the protocol will be permitted with approval of the Principal Investigator.

External sites will submit a master deviation log to the Coordinating Center monthly by email, at vrami2@jhu.edu and tlawre18@jhmi.edu.

10 Data Management

10.1 Data Collection

Data will be collected and reported on either paper case report forms (CRF) or through an internal data monitoring system, which will be determined prior to the start of the study.

10.2 Analyses and Reporting

Data Submission

Data and/or completed case report forms must be transmitted electronically using the study web based system provided by Johns Hopkins (CRMS). Data will be completed and submitted weekly for cycle 1 and monthly for each subsequent cycle. Case report forms will be provided to participating sites by the Coordinating Center.

Data will be analyzed and reported after the trial has completed accrual. All subsequent data collected will be analyzed and reported in a follow-up clinical report.

10.3 Clinical Trial Monitoring

The SKCCC Compliance Monitoring Program will provide external monitoring for JHU-affiliated sites in accordance with SKCCC DSMP (Version 6.0, 02/21/2019). The SMC Subcommittee will determine the level of patient safety risk and level/frequency of monitoring. Eligibility for all sites will be monitored by the Protocol Chair. The IND Sponsor and PI at each site shall internally monitor the progress of the trial, including review and confirmation of all safety/treatment-related outcomes, response assessments, safety reports and/or any related source documentation. Additional data and safety monitoring oversight will also be performed by the SKCCC Safety Monitoring Committee (SMC - as defined in the DSMP). External monitoring will occur according to the following guidelines:

Johns Hopkins SKCCC: The protocol will be monitored externally by the SKCCC CRO in accordance with SKCCC guidelines. Trial monitoring and reporting will be done through the Safety Monitoring Committee (SMC) at SKCCC.

Participating site(s): The protocol will be monitored by the internal CRO at each site. A report of the reviews will be submitted to the Johns Hopkins principal investigator and SKCCC CRO.

Authorized representatives of the Coordinating Center may visit the satellite sites to perform audits or inspections, including source data verification. The purpose of these audits or inspections is to systematically and independently examine all trial-related activities and documents to determine whether these activities were conducted and data were recorded, analyzed, and accurately reported according to the protocol, Good Clinical Practice (GCP), and any applicable regulatory requirements.

Dr. Azad will be holding the IND for this study. She will comply with all regulated reporting requirements to the FDA.

10.4 Investigator Responsibilities

Investigator responsibilities are set out in the ICH guideline for Good Clinical Practice (GCP) and in the US Code of Federal Regulations.

Investigators must enter study data onto CRFs or other data collection system. The Investigator will permit study-related audits by Celgene or its representatives, IRB/EC review, and regulatory

inspection(s) (e.g., FDA, EMEA, TPP), providing direct access to the facilities where the study took place, to source documents, to CRFs, and to all other study documents.

The Investigator, or a designated member of the Investigator's staff, must be available at some time during audits to review data and resolve any queries and to allow direct access to the subject's records (e.g., medical records, office charts, hospital charts, and study related charts) for source data verification. The data collection must be completed prior to each visit and be made available to the Celgene representative so that the accuracy and completeness may be checked.

10.5 Safety Meetings

Monthly teleconferences will be scheduled to include the PI/Sponsor, site Principal Investigators, Coordinating Center representatives, Site representatives and Celgene representatives (as appropriate). During these meetings, the Investigators shall provide the Coordinating Center with study progress updates. The Investigators will provide a summary of key points from the weekly meetings with a focus on safety of the protocol participants, enrollment status, and progress of data for objectives. In addition, Celgene will provide safety and applicable program updates to the Sponsor.

11 Biostatistical Analysis

11.1 Study Design/Endpoints

This trial is a phase II trial with the primary endpoint to demonstrate an increase in progression free survival in the CC-486 arm (Arm A) as compared to the observational arm (Arm B). Secondary endpoints include determining the response rate to chemotherapy, overall survival, and hypomethylation seen in the CC-486 arm as compared to the observational arm.

11.2 Study Populations

11.2.1 Efficacy Evaluable Population

The primary comparisons will include all randomized patients according to the assigned treatment groups (i.e. 'as randomized'). Secondary sensitivity analyses will examine the subset of patients receiving at least 1 dose of study drug as well as the subset of patients receiving at least 1 dose of chemotherapy. Comparisons of individuals based upon treatment received will also be examined as a sensitivity analysis.

11.2.2 Methylation Analysis Population

The methylation analysis population will consist of all subjects with any evaluable methylation results including plasma and/or tumor biopsies.

11.3 Statistical Methodology

11.3.1 Efficacy Analyses

Progression free survival is defined as the time from randomization until visible recurrence on any imaging modality, a confirmed biopsy, or death. Individuals lost to follow-up prior to having an event will be censored at the time of the last scan or biopsy. In the case of an equivocal scan reading, the patient will be re-imaged on schedule, if clinically appropriate, and if subsequent re-imaging confirms recurrence, the event will be considered to have occurred at the first time point when a related abnormality was noted. Overall survival is defined as the time from enrollment until death. Individuals lost to follow-up prior to death will be censored at the time of last physician contact.

Time to event outcomes, including the primary endpoint progression free survival and overall survival, will be summarized using Kaplan-Meier estimates of the survival function. From these estimates, median time to event and one-year survival proportions with 95% confidence intervals will be extracted. Cox proportional hazards models will be used to compare the two treatment arms and to evaluate and adjust for potential prognostic factors (e.g. nodal status, microscopic margins, increase in CA19-9, therapy). The number and type of responses to chemotherapy will be tabulated for each group. Logistic regression will be used to compare the response rate between the groups and to identify risk factors associated with response. Results will be considered statistically significant for p-values < 0.05.

11.3.2 Methylation Analyses

The correlative secondary studies will not affect sample size and are exploratory in nature only and are meant for hypothesis generation for further studies. Summary statistics (e.g. means, standard errors) and plots will be used to describe the pharmacodynamic endpoints at each time point (resection and relapse) as well as the change over time. T-tests will be used to assess the differences in the change in pharmacodynamic outcomes between those with and without CC486 exposure. These changes may also be quantified as a binary variable (response vs. no response) in which case the comparisons will be made using Fisher's exact test. Modeling approaches, which allow for the assessment of multiple variables, will also be considered for both the continuous outcomes (linear regression) and binary outcomes (logistic regression). A generalized estimating equation approach will also be considered to alleviate the impact of missing data on the inference.

11.3.3 Quality of life analysis

Quality of life will be assessed at screening and/or cycle 1 day 1 and then every 3 cycles thereafter (cycles 4, 7 and 10) using the Functional Assessment of Chronic Illness Therapy Hepatobiliary Cancer (FACIT-Hep) questionnaire. The hepatobiliary, general function, and subscale scores will

be will be graded according to the guidelines provided at http://www.facit.org. Summary statistics (e.g. means, medians, ranges) and graphical techniques (e.g. box-plots over time) will be used to summarize the patterns of scores over time and assess whether or not transformations are necessary. Comparisons of the differences in the change in scores over time between treatment groups will be performed using generalize estimating equations (GEE). Sensitivity analyses will be performed to assess the impact of missing data including best and worst case single imputation, multiple imputation. One issue when assessing quality of life is how to handle scores for individuals who have died. Some questionnaires (e.g. Stroke modified Rankin Score) include death as a value on the scale; however, this is not the case for the FACIT questionnaires. We will assess the impact of missing data due to death by assessing the worst category score for those who have died.

11.3.4 Sample Size

The anecdotal time to progression without treatment is approximately 4 to 6 months. A feasible goal of 60 patients (30 in each arm) will provide 80% power to identify a 64% improvement in the median PFS from the null rate of 6 months assuming a one-sided type 1 error rate of 0.20, 24 months of accrual and a minimum of 12 months of follow-up while allowing for 10% loss to follow-up (protocol accruing for 6 months at the time of submission.

1-sided Type I error rate	Detectable HR (N = 60)
0.05	2.12
0.10	1.89
0.15	1.74
0.20	1.64

11.3.5 Safety Monitoring

Although the dose level is below the maximally tolerated dose, we will monitor the rates of grade 3/4 toxicity for individuals treated with CC-486. We would expect to observe grade 3/4 toxicities in 15% of patients. The goal is to ensure that the percentage of grade 3/4 toxicities remains below 30%. A Bayesian stopping guideline has been developed. The prior was chosen to be a Beta (1, 4), representing a 20% toxicity rate, which is at the high end of our expected value. The guidelines will recommend that the therapy be re-evaluated if the posterior probability of toxicity exceeding 30% is greater than 65%. Table 11.3.5.1 below summarizes the stopping guideline boundaries.

Table 11.3.5.1

Cohort size	Observed number of toxicities that trigger re-evaluation
6-9	4
10-12	5
13-15	6
16-18	7
19-21	8
22-24	9
25-27	10
28-31	11

The probability of triggering the stopping guidelines was assessed for a range of underlying toxicity rates based upon simulations with 5000 replicates (Table 11.3.5.2 below). In the expected range of toxicity, the probability of stopping early was less than 4.8%.

Table 11.3.5.2

True underlying toxicity rate	Probability of triggering stopping guideline boundaries
10%	1.2%
15%	4.8%
20%	14.1%
25%	32.2%
30%	54.1%
40%	89.6%
50%	99.1%

11.3.6 Futility Analysis

An interim futility analysis will be performed once 50% of the expected progressions have occurred. The conditional power will be calculated for the following three scenarios for the unobserved data:

- Null hypothesis: Assumes no difference between the two treatment arms, i.e. the median PFS in both groups is 6 months.

- Alternative hypothesis: Assumes that the originally hypothesized rates (6 months vs 9.84 months) will be observed.
- Current pattern: Assumes that the PFS observed for each group in the initial cohort will be observed for the remaining patients.

The decision to stop or continue will be based upon the currently observed pattern with the null and alternative hypothesis patterns providing an estimate of the range of possible values. Since the cost of continuing (in terms of safety, resources and monetary cost) is low relative to the information to be gained, a low threshold for continuing was selected. We will stop the trial and conclude futility if the conditional power is 30% or less. Otherwise, the trial will continue. No interim efficacy analysis is planned due to the high cost of type I error spending associated with an interim efficacy analysis.

12 Regulatory Considerations

12.1 Institutional Review Board/Ethics Committee approval

The protocol for this study has been designed in accordance with the general ethical principles outlined in the Declaration of Helsinki. The review of this protocol by the IRB/EC and the performance of all aspects of the study, including the methods used for obtaining informed consent, must also be in accordance with principles enunciated in the declaration, as well as ICH Guidelines, Title 21 of the Code of Federal Regulations (CFR), Part 50 Protection of Human Subjects and Part 56 Institutional Review Boards.

The Investigator will be responsible for preparing documents for submission to the relevant IRB/EC and obtaining written approval for this study. The approval will be obtained prior to the initiation of the study.

The approval for both the protocol and informed consent must specify the date of approval, protocol number and version, or amendment number. Any amendments to the protocol after receipt of IRB/EC approval must be submitted by the Investigator to the IRB/EC for approval. The Investigator is also responsible for notifying the IRB/EC of any serious deviations from the protocol, or anything else that may involve added risk to subjects.

Any advertisements used to recruit subjects for the study must be reviewed and approved by the IRB/EC prior to use.

12.2 Informed Consent

The Investigator must obtain informed consent of a subject or his/her designee prior to any study related procedures as per GCPs as set forth in the CFR and ICH guidelines.

Documentation that informed consent occurred prior to the subject's entry into the study and the informed consent process should be recorded in the subject's source documents. The original consent form signed and dated by the subject and by the person consenting the subject prior to the subject's entry into the study must be maintained in the Investigator's study files.

12.3 Subject Confidentiality

Celgene affirms the subject's right to protection against invasion of privacy. In compliance with United States federal regulations, Celgene requires the Investigator to permit representatives of Celgene Corporation and, when necessary, representatives of the FDA or other regulatory authorities to review and/or copy any medical records relevant to the study in accordance with local laws.

Should direct access to medical records require a waiver or authorization separate from the subject's statement of informed consent, it is the responsibility of the Investigator to obtain such permission in writing from the appropriate individual.

12.4 Study Records Requirements

The Investigator must ensure that the records and documents pertaining to the conduct of the study and the distribution of the protocol therapy, that is copies of CRFs and source documents (original documents, data, and records [e.g., hospital records; clinical and office charts; laboratory notes; memoranda; subject's diaries or evaluation checklists; SAE reports, pharmacy dispensing records; recorded data from automated instruments; copies or transcriptions certified after verification as being accurate copies; microfiches; photographic negatives, microfilm, or magnetic media; x-rays; subject files; and records kept at the pharmacy, at the laboratories, and at medico-technical departments involved in the clinical study; documents regarding subject treatment and drug accountability; original signed informed consents, etc.]) be retained by the Investigator for as long as needed to comply with national and international regulations (generally 2 years after discontinuing clinical development or after the last marketing approval). The Investigator agrees to adhere to the document/records retention procedures by signing the protocol.

12.5 Premature Discontinuation of Study

The Principal Investigator, institution and Celgene have the right to discontinue this study at any time for reasonable medical or administrative reasons. Possible reasons for termination of the study could be but are not limited to:

- Unsatisfactory enrollment with respect to quantity or quality.
- Inaccurate or incomplete data collection.
- Falsification of records.
- Failure to adhere to the study protocol.

Any possible premature discontinuation would be documented adequately with reasons being stated, and information would have to be issued according to local requirements (e.g., IRB/EC, regulatory authorities, etc.).

13 References

1. Siegel R, Ward E, Brawley O, Jemal A. Cancer statistics, 2011: The impact of eliminating socioeconomic and racial disparities on premature cancer deaths. *CA Cancer J Clin* 2011; **61**(4): 212-36.
2. Bunger S, Laubert T, Roblick UJ, Habermann JK. Serum biomarkers for improved diagnostic of pancreatic cancer: a current overview. *J Cancer Res Clin Oncol* 2011; **137**(3): 375-89.
3. Sharma C, Eltawil KM, Renfrew PD, Walsh MJ, Molinari M. Advances in diagnosis, treatment and palliation of pancreatic carcinoma: 1990-2010. *World J Gastroenterol* 2011; **17**(7): 867-97.
4. Van Laethem JL, Hammel P, Mornex F, et al. Adjuvant gemcitabine alone versus gemcitabine-based chemoradiotherapy after curative resection for pancreatic cancer: a randomized EORTC-40013-22012/FFCD-9203/GERCOR phase II study. *J Clin Oncol* 2010; **28**(29): 4450-6.
5. Vincent A, Herman J, Schulick R, Hruban RH, Goggins M. Pancreatic cancer. *Lancet* 2011; **378**(9791): 607-20.
6. Edge SB, Compton CC. The American Joint Committee on Cancer: the 7th edition of the AJCC cancer staging manual and the future of TNM. *Ann Surg Oncol* 2010; **17**(6): 1471-4.
7. Tomlinson JS, Jain S, Bentrem DJ, et al. Accuracy of staging node-negative pancreas cancer: a potential quality measure. *Arch Surg* 2007; **142**(8): 767-23; discussion 73-4.
8. Pawlik TM, Gleisner AL, Cameron JL, et al. Prognostic relevance of lymph node ratio following pancreaticoduodenectomy for pancreatic cancer. *Surgery* 2007; **141**(5): 610-8.
9. Yeo CJ, Cameron JL, Lillemoe KD, et al. Pancreaticoduodenectomy with or without distal gastrectomy and extended retroperitoneal lymphadenectomy for periampullary adenocarcinoma, part 2: randomized controlled trial evaluating survival, morbidity, and mortality. *Ann Surg* 2002; **236**(3): 355-66; discussion 66-8.

10. Winter JM, Cameron JL, Campbell KA, et al. 1423 pancreaticoduodenectomies for pancreatic cancer: A single-institution experience. *J Gastrointest Surg* 2006; **10**(9): 1199-210; discussion 210-1.
11. Takahashi H, Ohigashi H, Ishikawa O, et al. Perineural invasion and lymph node involvement as indicators of surgical outcome and pattern of recurrence in the setting of preoperative gemcitabine-based chemoradiation therapy for resectable pancreatic cancer. *Ann Surg* 2012; **255**(1): 95-102.
12. Katz MH, Wang H, Fleming JB, et al. Long-term survival after multidisciplinary management of resected pancreatic adenocarcinoma. *Ann Surg Oncol* 2009; **16**(4): 836-47.
13. Showalter TN, Winter KA, Berger AC, et al. The influence of total nodes examined, number of positive nodes, and lymph node ratio on survival after surgical resection and adjuvant chemoradiation for pancreatic cancer: a secondary analysis of RTOG 9704. *Int J Radiat Oncol Biol Phys* 2011; **81**(5): 1328-35.
14. Garcea G, Dennison AR, Pattenden CJ, Neal CP, Sutton CD, Berry DP. Survival following curative resection for pancreatic ductal adenocarcinoma. A systematic review of the literature. *JOP* 2008; **9**(2): 99-132.
15. Schmidt CM, Glant J, Winter JM, et al. Total pancreatectomy (R0 resection) improves survival over subtotal pancreatectomy in isolated neck margin positive pancreatic adenocarcinoma. *Surgery* 2007; **142**(4): 572-8; discussion 8-80.
16. Tseng JF, Raut CP, Lee JE, et al. Pancreaticoduodenectomy with vascular resection: margin status and survival duration. *J Gastrointest Surg* 2004; **8**(8): 935-49; discussion 49-50.
17. Ferrone CR, Finkelstein DM, Thayer SP, Muzikansky A, Fernandez-delCastillo C, Warshaw AL. Perioperative CA19-9 levels can predict stage and survival in patients with resectable pancreatic adenocarcinoma. *J Clin Oncol* 2006; **24**(18): 2897-902.
18. Berger AC, Garcia M, Jr., Hoffman JP, et al. Postresection CA 19-9 predicts overall survival in patients with pancreatic cancer treated with adjuvant chemoradiation: a prospective validation by RTOG 9704. *J Clin Oncol* 2008; **26**(36): 5918-22.
19. Lomberk GA. Epigenetic silencing of tumor suppressor genes in pancreatic cancer. *J Gastrointest Cancer* 2011; **42**(2): 93-9.
20. McCabe MT, Brandes JC, Vertino PM. Cancer DNA methylation: molecular mechanisms and clinical implications. *Clin Cancer Res* 2009; **15**(12): 3927-37.
21. Grzenda A, Ordog T, Urrutia R. Polycomb and the emerging epigenetics of pancreatic cancer. *J Gastrointest Cancer* 2011; **42**(2): 100-11.
22. Sherr CJ. Principles of tumor suppression. *Cell* 2004; **116**(2): 235-46.
23. Koorstra JB, Hustinx SR, Offerhaus GJ, Maitra A. Pancreatic carcinogenesis. *Pancreatology* 2008; **8**(2): 110-25.

24. Issa JP, Kantarjian HM. Targeting DNA methylation. *Clin Cancer Res* 2009; **15**(12): 3938-46.
25. Silverman LR, Demakos EP, Peterson BL, et al. Randomized controlled trial of azacitidine in patients with the myelodysplastic syndrome: a study of the cancer and leukemia group B. *J Clin Oncol* 2002; **20**(10): 2429-40.
26. Fenaux P, Mufti GJ, Hellstrom-Lindberg E, et al. Azacitidine prolongs overall survival compared with conventional care regimens in elderly patients with low bone marrow blast count acute myeloid leukemia. *J Clin Oncol* 2010; **28**(4): 562-9.
27. Lubbert M, Suciu S, Baila L, et al. Low-dose decitabine versus best supportive care in elderly patients with intermediate- or high-risk myelodysplastic syndrome (MDS) ineligible for intensive chemotherapy: final results of the randomized phase III study of the European Organisation for Research and Treatment of Cancer Leukemia Group and the German MDS Study Group. *J Clin Oncol* 2011; **29**(15): 1987-96.
28. Fenaux P, Mufti GJ, Hellstrom-Lindberg E, et al. Efficacy of azacitidine compared with that of conventional care regimens in the treatment of higher-risk myelodysplastic syndromes: a randomised, open-label, phase III study. *Lancet Oncol* 2009; **10**(3): 223-32.
29. Garcia-Manero G. Myelodysplastic syndromes: 2011 update on diagnosis, riskstratification, and management. *Am J Hematol* 2011; **86**(6): 490-8.
30. Garcia-Manero G, Stoltz ML, Ward MR, Kantarjian H, Sharma S. A pilot pharmacokinetic study of oral azacitidine. *Leukemia* 2008; **22**(9): 1680-4.
31. Garcia-Manero G, Gore SD, Cogle C, et al. Phase I study of oral azacitidine in myelodysplastic syndromes, chronic myelomonocytic leukemia, and acute myeloid leukemia. *J Clin Oncol* 2011; **29**(18): 2521-7.
32. Hui AB, Lo KW, Kwong J, et al. Epigenetic inactivation of TSLC1 gene in nasopharyngeal carcinoma. *Mol Carcinog* 2003; **38**(4): 170-8.
33. Chan AS, Tsui WY, Chen X, et al. Downregulation of ID4 by promoter hypermethylation in gastric adenocarcinoma. *Oncogene* 2003; **22**(44): 6946-53.
34. Li M, Zhao ZW. Clinical implications of mismatched repair gene promoter methylation in pancreatic cancer. *Med Oncol* 2011.
35. Yang H, Lu X, Qian J, et al. Imprinted tumor suppressor gene ARHI induces apoptosis correlated with changes in DNA methylation in pancreatic cancer cells. *Mol Med Report* 2010; **3**(4): 581-7.
36. Kuroki T, Tajima Y, Kanematsu T. Role of hypermethylation on carcinogenesis in the pancreas. *Surg Today* 2004; **34**(12): 981-6.
37. Cai HH, Sun YM, Miao Y, et al. Aberrant methylation frequency of TNFRSF10C promoter in pancreatic cancer cell lines. *Hepatobiliary Pancreat Dis Int* 2011; **10**(1): 95-100.

38. Ramachandran K, Miller H, Gordian E, Rocha-Lima C, Singal R. Methylation-mediated silencing of TMS1 in pancreatic cancer and its potential contribution to chemosensitivity. *Anticancer Res* 2010; **30**(10): 3919-25.
39. Gupta S, Pramanik D, Mukherjee R, et al. Molecular determinants of retinoic acid sensitivity in pancreatic cancer. *Clin Cancer Res* 2012; **18**(1): 280-9.
40. Oettle H, Post S, Neuhaus P, et al. Adjuvant chemotherapy with gemcitabine vs observation in patients undergoing curative-intent resection of pancreatic cancer: a randomized controlled trial. *JAMA* 2007; **297**(3): 267-77.
41. Neoptolemos JP, Stocken DD, Friess H, et al. A randomized trial of chemoradiotherapy and chemotherapy after resection of pancreatic cancer. *N Engl J Med* 2004; **350**(12): 1200-10.
42. Neoptolemos JP, Stocken DD, Bassi C, et al. Adjuvant chemotherapy with fluorouracil plus folinic acid vs gemcitabine following pancreatic cancer resection: a randomized controlled trial. *JAMA* 2010; **304**(10): 1073-81.
43. Benson AB, 3rd, Ajani JA, Catalano RB, et al. Recommended guidelines for the treatment of cancer treatment-induced diarrhea. *J Clin Oncol* 2004; **22**(14): 2918-26.
44. Eisenhauer EA, Therasse P, Bogaerts J, et al. New response evaluation criteria in solid tumours: revised RECIST guideline (version 1.1). *Eur J Cancer* 2009; **45**(2): 228-47.

14 Appendices

Appendix A: ECOG Performance Status Scale

SCORE	DESCRIPTION
**0**	Fully active, able to carry on all pre-disease performance without restriction.
**1**	Restricted in physically strenuous activity but ambulatory and able to carry out work of a light or sedentary nature, e.g., light house work, office work.
**2**	Ambulatory and capable of all self-care but unable to carry out any work activities. Up and about more than 50% of waking hours.
**3**	Capable of only limited self-care, confined to bed or chair more than 50% of waking hours.
**4**	Completely disabled. Cannot carry on any self-care. Totally confined to bed or chair.
**5**	Dead.

Appendix B: Medication Diary

J12138
Medication Diary – CC-486
Cycle Number __________
Day 1 Date _____________
Name_______________________(initials acceptable) ID_____________________

INSTRUCTIONS:				
1. Complete one form per cycle of therapy.<br>2. Take your dose of the study drug days 1 through 21 of each 28 day cycle. CC-486 may be taken on an empty stomach or with food. The tablets should be taken with about 1 cup (240mL) of water. You will take 3 tablets each day (or fewer if you have been dose reduced by your physician). You should swallow the tablets whole. Do not chew, crush, or break the tablets. If a dose of CC-486 is missed, it should be taken as soon as possible on the same day. If it is missed for the entire day, it should <u>not</u> be made up. If the patient vomits after a dose is taken, no more subsequent tablets should be administered.<br>3. Record the date, the number of tablets of you took, and when you took them.<br>4. If you have any comments or notice any side effects, please record them in the Comments section.<br>5. Please return the forms to your physician/research nurse prior to the next cycle of therapy.<br>6. **On clinic days-please wait to take your medication until after you have been seen by the RN or Doctor**				
Day	Date	What time was the dose taken?	# of tablets	Comments
1				
2				
3				
4				
5				
6				
7				
8				
9				
10				
11				

12				
13				
14				
15				
16				
17				
18				
19				
20				
21				
To be completed by research study staff:<br>1. Date protocol treatment was started<br>____<br>2. Date subject was removed from protocol<br>____<br>3. Planned total daily dose<br>____<br>4. Total number of pills taken this cycle<br>____<br>5. Research Study Staff's Signature:<br>____				

Participant's signature ____________________________________ Date _______________

Appendix C: Recommendations for Management of Treatment-Induced Diarrhea (for CC-486)

The following guidelines are based on a publication by Benson *et al.* in the *Journal of Clinical Oncology*[43] .

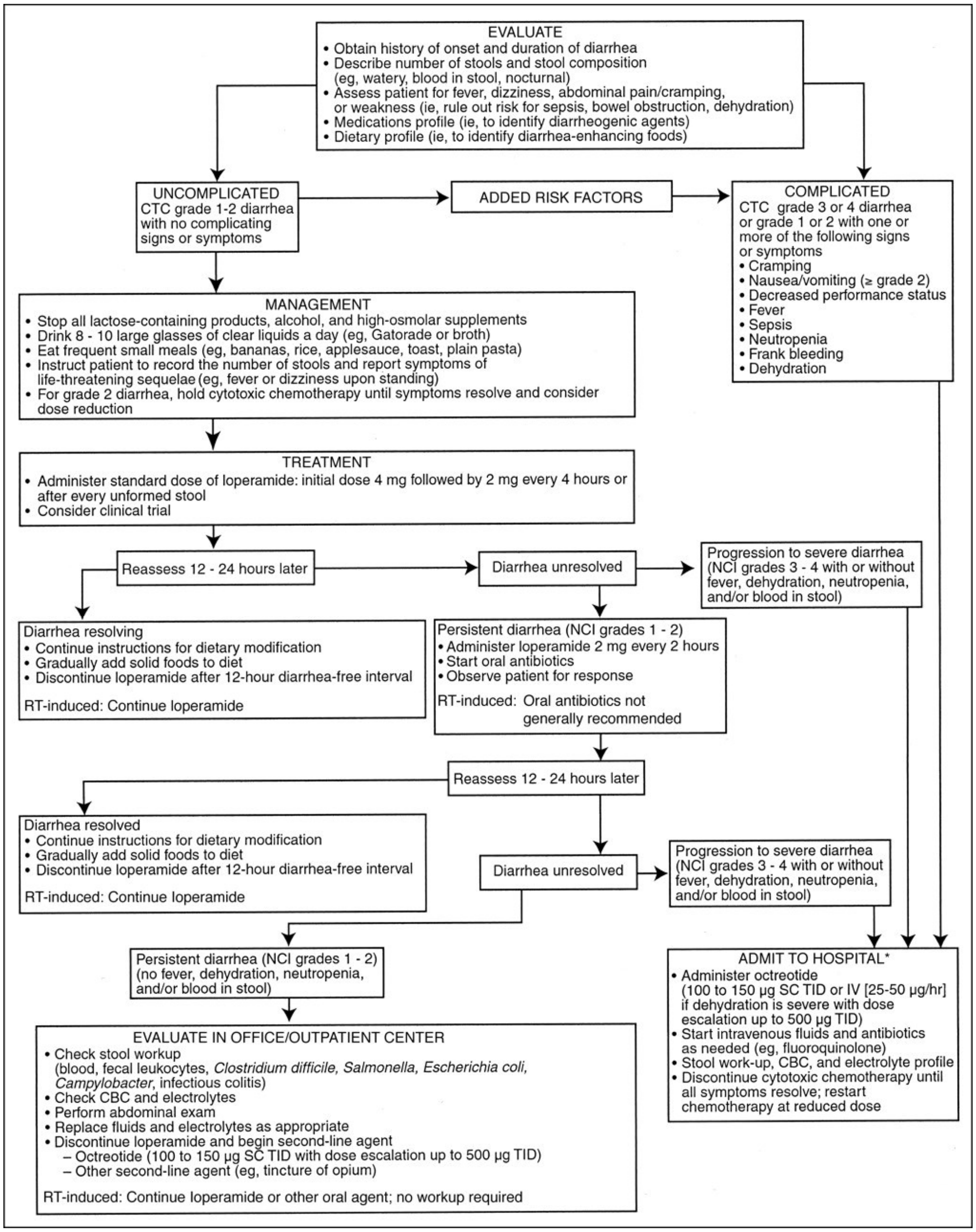

Appendix D: FACT – Hep (Version 4)

FACT-Hep (Version 4)

Below is a list of statements that other people with your illness have said are important. **Please circle or mark one number per line to indicate your response as it applies to the <u>past 7 days</u>.**

	PHYSICAL WELL-BEING	Not at all	A little bit	Some-what	Quite a bit	Very much
GP1	I have a lack of energy	0	1	2	3	4
GP2	I have nausea	0	1	2	3	4
GP3	Because of my physical condition, I have trouble meeting the needs of my family	0	1	2	3	4
GP4	I have pain	0	1	2	3	4
GP5	I am bothered by side effects of treatment	0	1	2	3	4
GP6	I feel ill	0	1	2	3	4
GP7	I am forced to spend time in bed	0	1	2	3	4

	SOCIAL/FAMILY WELL-BEING	Not at all	A little bit	Some-what	Quite a bit	Very much
GS1	I feel close to my friends	0	1	2	3	4
GS2	I get emotional support from my family	0	1	2	3	4
GS3	I get support from my friends	0	1	2	3	4
GS4	My family has accepted my illness	0	1	2	3	4
GS5	I am satisfied with family communication about my illness	0	1	2	3	4
GS6	I feel close to my partner (or the person who is my main support)	0	1	2	3	4
Q1	*Regardless of your current level of sexual activity, please answer the following question. If you prefer not to answer it, please mark this box* ☐ *and go to the next section.*					
GS7	I am satisfied with my sex life	0	1	2	3	4

English (Universal)
Copyright 1987, 1997

16 November 2007

FACT-Hep (Version 4)

Please circle or mark one number per line to indicate your response as it applies to the <u>past 7 days</u>.

	**<u>EMOTIONAL WELL-BEING</u>**	**Not at all**	**A little bit**	**Some-what**	**Quite a bit**	**Very much**
GE1	I feel sad	0	1	2	3	4
GE2	I am satisfied with how I am coping with my illness	0	1	2	3	4
GE3	I am losing hope in the fight against my illness	0	1	2	3	4
GE4	I feel nervous	0	1	2	3	4
GE5	I worry about dying	0	1	2	3	4
GE6	I worry that my condition will get worse	0	1	2	3	4

	**<u>FUNCTIONAL WELL-BEING</u>**	**Not at all**	**A little bit**	**Some-what**	**Quite a bit**	**Very much**
GF1	I am able to work (include work at home)	0	1	2	3	4
GF2	My work (include work at home) is fulfilling	0	1	2	3	4
GF3	I am able to enjoy life	0	1	2	3	4
GF4	I have accepted my illness	0	1	2	3	4
GF5	I am sleeping well	0	1	2	3	4
GF6	I am enjoying the things I usually do for fun	0	1	2	3	4
GF7	I am content with the quality of my life right now	0	1	2	3	4

English (Universal)
Copyright 1987, 1997

16 November 2007
Page 2 of 3

FACT-Hep (Version 4)

Please circle or mark one number per line to indicate your response as it applies to the <u>past 7 days</u>.

	**<u>ADDITIONAL CONCERNS</u>**	**Not at all**	**A little bit**	**Some-what**	**Quite a bit**	**Very much**
C1	I have swelling or cramps in my stomach area	0	1	2	3	4
C2	I am losing weight	0	1	2	3	4
C3	I have control of my bowels	0	1	2	3	4
C4	I can digest my food well	0	1	2	3	4
C5	I have diarrhea (diarrhoea)	0	1	2	3	4
C6	I have a good appetite	0	1	2	3	4
Hep 1	I am unhappy about a change in my appearance	0	1	2	3	4
CNS 7	I have pain in my back	0	1	2	3	4
Cx6	I am bothered by constipation	0	1	2	3	4
HI7	I feel fatigued	0	1	2	3	4
An7	I am able to do my usual activities	0	1	2	3	4
Hep 2	I am bothered by jaundice or yellow color to my skin	0	1	2	3	4
Hep 3	I have had fevers (episodes of high body temperature)	0	1	2	3	4
Hep 4	I have had itching	0	1	2	3	4
Hep 5	I have had a change in the way food tastes	0	1	2	3	4
Hep 6	I have had chills	0	1	2	3	4
HN 2	My mouth is dry	0	1	2	3	4
Hep 8	I have discomfort or pain in my stomach area	0	1	2	3	4

English (Universal)
Copyright 1987, 1997

16 November 2007
Page 3 of 3
