# Supplementary material for: A randomized, phase II trial of oral azacitidine (CC-486) in patients with resected pancreatic adenocarcinoma at high risk for recurrence
Source: Clin Epigenetics. 2022 Dec 3;14:166. doi: 10.1186/s13148-022-01367-8 (PMC9719150; doi:10.1186/s13148-022-01367-8)
Supplement: Supplementary file 2 — Additional file 2. Supplemental Materials. Fig S1: Global change in methylation after treatment. [a] The following violin plots show change in methylation after treatment, compared to before. Change is calculated as the simple difference between methylation levels after and before treatment. [b] The relationship between FDR and change in beta is shown in the following volcano plot. The red lines indicate a change in beta of 0.10. Table S1: Association between confirmed progression free survival and characteristics of interest. Table S2: Characteristics of patients received metastatic chemotherapy stratified by study treatment group. Table S3: Best Response to Systemic Therapy in Advanced Disease Setting. Table S4: Association between metastatic progression free survival and characteristics among participants receiving chemotherapy in the advanced disease setting. [file 13148_2022_1367_MOESM2_ESM.docx]

**Supplemental Materials**

**Contents**

Appendix A: Study Protocol [separate attachment]

Appendix B [Fig S1]: Global change in methylation after treatment.

Appendix C [Table S1]: Association between confirmed progression free survival and characteristics of interest

Appendix D [Table S2]: Characteristics of patients received metastatic chemotherapy stratified by study treatment group

Appendix E [Table S3]: Best Response to Systemic Therapy in Advanced Disease Setting

Appendix F [Table S4]: Association between metastatic progression free survival and characteristics among participants receiving chemotherapy in the advanced disease setting.

**Fig S1:** Global change in methylation after treatment

[a] The following violin plots show change in methylation after treatment, compared to before. Change is calculated as the simple difference between methylation levels after and before treatment. [b] The relationship between FDR and change in beta is shown in the following volcano plot. The red lines indicate a change in beta of 0.10.

**
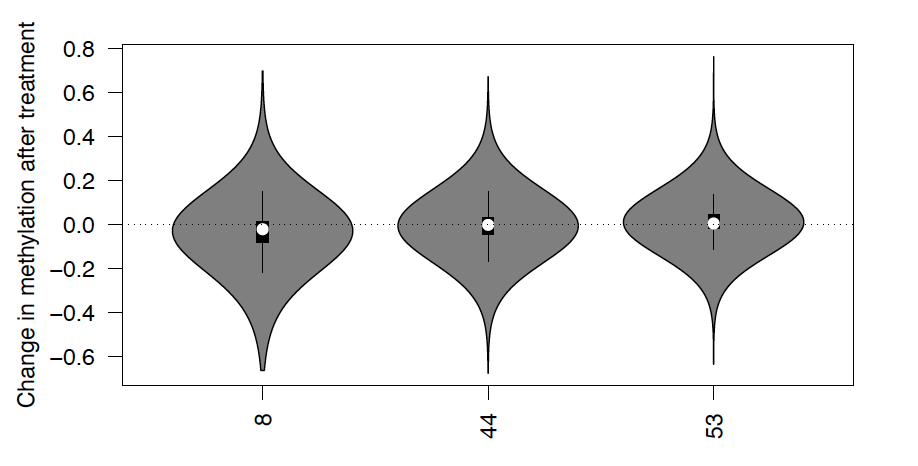
**

**
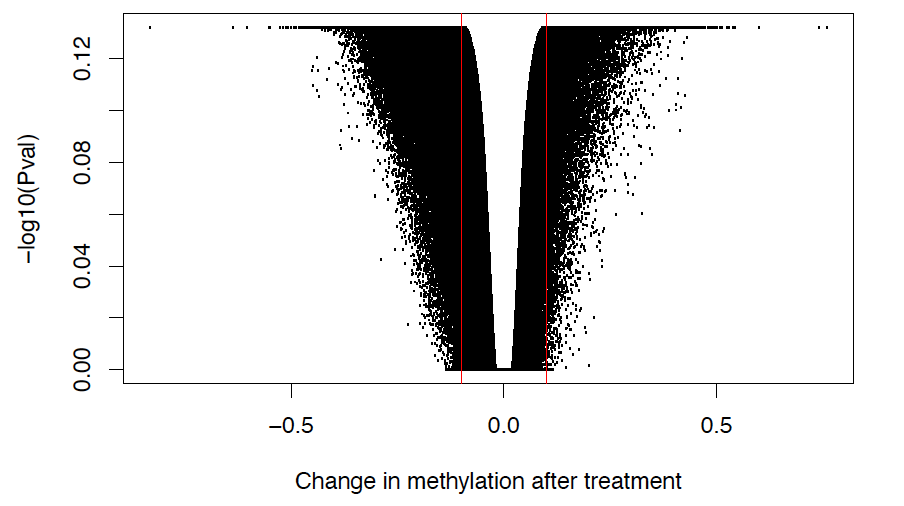
**

**Table S1:** Association between confirmed progression free survival and characteristics of interest

| **Characteristic** | **N** | **Events** | **Median** | **(95% CI)** | **2-year OS %** | **(95% CI)** | **Hazard Ratio** | **(95% CI)** | ***p*** |
| --- | --- | --- | --- | --- | --- | --- | --- | --- | --- |
| Overall | 48 | 34 | 9.2 | (5.2-18.7) | 44 | (28-59) |  |  |  |
|  |  |  |  |  |  |  |  |  |  |
| Assigned treatment |  |  |  |  |  |  |  |  |  |
| Observation | 25 | 16 | 8.9 | (3.4-24.6) | 50 | (27-69) | 1 |  | 0.85 |
| CC-486 | 23 | 18 | 9.2 | (4.1-20.9) | 38 | (18-58) | 0.94 | (0.46-1.87) |  |
|  |  |  |  |  |  |  |  |  |  |
| Eligibility: CA 19-9 |  |  |  |  |  |  |  |  |  |
| No | 23 | 18 | 13.1 | (5.9-24.6) | 52 | (30-71) | 1 |  | 0.31 |
| Yes | 25 | 16 | 5.3 | (2.0-42.6) | 35 | (15-57) | 1.42 | (0.72-2.81) |  |
|  |  |  |  |  |  |  |  |  |  |
| Eligibility: R1 |  |  |  |  |  |  |  |  |  |
| No | 38 | 26 | 9.2 | (5.1-18.7) | 45 | (27-62) | 1 |  | 0.25 |
| Yes | 10 | 8 | 8.7 | (2.2-47.6) | 40 | (12-68) | 0.58 | (0.23-1.47) |  |
|  |  |  |  |  |  |  |  |  |  |
| Eligibility: Positive lymph nodes |  |  |  |  |  |  |  |  |  |
| No | 13 | 9 | 3.3 | (1.8-42.6) | 39 | (12-65) | 1 |  | 0.59 |
| Yes | 35 | 25 | 9.3 | (5.4-20.9) | 46 | (28-63) | 0.81 | (0.37-1.75) |  |
|  |  |  |  |  |  |  |  |  |  |
| Age (years) |  |  |  |  |  |  |  |  |  |
| Under 60 | 11 | 6 | 5.5 | (1.2-Inf) | 40 | (12- 68) | 1 |  | 0.58 |
| 60 or older | 37 | 28 | 9.3 | (5.2-18.7) | 45 | (27-62) | 1.29 | (0.53-3.12) |  |
|  |  |  |  |  |  |  |  |  |  |
| Gender |  |  |  |  |  |  |  |  |  |
| Male | 25 | 16 | 15.8 | (5.2-44.6) | 55 | (31-73) | 1 |  | 0.20 |
| Female | 23 | 18 | 6.4 | (3.4-13.1) | 33 | (14-54) | 1.56 | (0.78-3.09) |  |
|  |  |  |  |  |  |  |  |  |  |
| ECOG |  |  |  |  |  |  |  |  |  |
| 0 | 39 | 26 | 9.2 | (5.4-24.6) | 44 | (26-60) | 1 |  | 0.15 |
| 1 | 9 | 8 | 6.0 | (0.3-15.8) | 44 | (13-72) | 1.82 | (0.80-4.15) |  |
|  |  |  |  |  |  |  |  |  |  |
| CA 19-9 at randomization |  |  |  |  |  |  |  |  |  |
| < 100 | 41 | 29 | 11.0 | (5.9-20.9) | 49 | (32-65) | 1 |  | < 0.001 |
| >=100 | 7 | 5 | 2.5 | (1.2-Inf) | 0 |  | 7.73 | (2.51-23.77) |  |
|  |  |  |  |  |  |  |  |  |  |
| Surgical resection margin status |  |  |  |  |  |  |  |  |  |
| R0 | 38 | 26 | 9.2 | (5.1-18.7) | 45 | (27-62) | 1 |  | 0.25 |
| R1 | 10 | 8 | 8.7 | (2.2-47.6) | 40 | (12-68) | 0.58 | (0.23-1.47) |  |
|  |  |  |  |  |  |  |  |  |  |
| Histologic grade |  |  |  |  |  |  |  |  |  |
| Well/moderately differentiated | 38 | 26 | 9.2 | (5.4-18.7) | 43 | (26-60) | 1 |  | 0.67 |
| Poorly differentiated | 10 | 8 | 6.0 | (1.2-44.6) | 44 | (13-72) | 1.19 | (0.53-2.67) |  |
|  |  |  |  |  |  |  |  |  |  |
| T stage |  |  |  |  |  |  |  |  |  |
| T1/T2 | 28 | 18 | 9.3 | (3.4-20.9) | 48 | (27-66) | 1 |  | 0.99 |
| T3/T4 | 20 | 16 | 9.2 | (5.1-24.6) | 39 | (17-61) | 1.00 | (0.50-2.00) |  |
|  |  |  |  |  |  |  |  |  |  |
| Positive lymph nodes |  |  |  |  |  |  |  |  |  |
| No | 13 | 9 | 3.3 | (1.8-42.6) | 39 | (12-65) | 1 |  | 0.59 |
| Yes | 35 | 25 | 9.3 | (5.4-20.9) | 46 | (28-63) | 0.81 | (0.37-1.75) |  |
|  |  |  |  |  |  |  |  |  |  |
| Lymphovascular invasion (LVI) |  |  |  |  |  |  |  |  |  |
| No | 16 | 11 | 11.0 | (2.2-20.9) | 43 | (18-67) | 1 |  | 0.93 |
| Yes | 27 | 20 | 9.2 | (5.1-24.9) | 47 | (26-66) | 1.04 | (0.49-2.18) |  |
|  |  |  |  |  |  |  |  |  |  |
| Perineural spread/invasion (PNS) |  |  |  |  |  |  |  |  |  |
| No | 6 | 4 | 2.3 | (1.2-Inf) | 22 | (0-62) | 1 |  | 0.63 |
| Yes | 42 | 30 | 9.3 | (5.4-20.9) | 47 | (30-62) | 0.77 | (0.26-2.25) |  |
|  |  |  |  |  |  |  |  |  |  |
| Pre-trial radiation therapy |  |  |  |  |  |  |  |  |  |
| None | 17 | 10 | 24.6 | (9.1-42.6) | 70 | (38-88) | 1 |  |  |
| Neo-adjuvant only | 11 | 8 | 6.2 | (1.8-11.0) | 20 | (3-49) | 2.03 | (0.78-5.24) | 0.14 |
| Adjuvant only | 20 | 16 | 5.5 | (2.5-13.5) | 38 | (17-59) | 1.59 | (0.70-3.59) | 0.26 |
|  |  |  |  |  |  |  |  |  |  |
| Pre-trial systemic therapy |  |  |  |  |  |  |  |  |  |
| Neo-adjuvant only or adjuvant only | 30 | 21 | 13.1 | (5.4-24.6) | 51 | (31-69) | 1 |  | 0.30 |
| Both neo-adjuvant and adjuvant | 18 | 13 | 6.2 | (1.8-20.9) | 32 | (11-55) | 1.46 | (0.71-3.00) |  |
|  |  |  |  |  |  |  |  |  |  |

**Table S2**: Characteristics of patients received metastatic chemotherapy stratified by study treatment group

| **Characteristic** | **Total**  **(N=26)** | **Maintenance Observation**  **(N=13)** | **Maintenance CC-486**  **(N=13)** | **P-value** |
| --- | --- | --- | --- | --- |
| Eligibility, N(%) |  |  |  |  |
| CA 19-9 | 12 (46%) | 7 (54%) | 5 (38%) | 0.7 |
| R1 | 6 (23%) | 1 (8%) | 5 (38%) | 0.16 |
| Node | 20 (77%) | 10 (77%) | 10 (77%) | > 0.99 |
|  |  |  |  |  |
| Age (years) |  |  |  |  |
| Median (Q1, Q3) | 66 (60, 72) | 71 (60, 75) | 64 (60, 68) | 0.16 |
| 60 or older, N (%) | 20 (77%) | 10 (77%) | 10 (77%) | > 0.99 |
| Sex |  |  |  |  |
| Female, N (%) | 11 (42%) | 5 (38%) | 6 (46%) | > 0.99 |
|  |  |  |  |  |
| ECOG at randomization, N (%) |  |  |  | > 0.99 |
| 0 | 20 (77%) | 10 (77%) | 10 (77%) |  |
| 1 | 6 (23%) | 3 (23%) | 3 (23%) |  |
|  |  |  |  |  |
| CA 19-9 at randomization |  |  |  |  |
| Median (Q1, Q3) | 31.6 (12.6, 78.3) | 42.7 (11.8, 83.9) | 31.0 (15.2, 54.3) | 0.90 |
| >= 100, N(%) | 4 (15%) | 2 (15%) | 2 (15%) | > 0.99 |
|  |  |  |  |  |
| Time from surgery to randomization (years) |  |  |  |  |
| Median (Q1, Q3) | 8.9 (7.8, 11.1) | 9.6 (7.9, 12.7) | 8.7 (7.0, 10.7) | 0.15 |
|  |  |  |  |  |
| Surgical resection margin status, N (%) |  |  |  | 0.16 |
| R0 | 20 (77%) | 12 (92%) | 8 (62%) |  |
| R1 | 6 (23%) | 1 (8%) | 5 (38%) |  |
|  |  |  |  |  |
| Histologic grade, N(%) |  |  |  | > 0.99 |
| Well/moderately differentiated | 22 (85%) | 11 (85%) | 11 (85%) |  |
| Poorly differentiated | 4 (15%) | 2 (15%) | 2 (15%) |  |
|  |  |  |  |  |
| T stage, N(%) |  |  |  | > 0.99 |
| T1/T2 | 17 (65%) | 9 (69%) | 8 (62%) |  |
| T3/T4 (involves celiac axis, SM or common hepatic artery) | 9 (35%) | 4 (31%) | 5 (38%) |  |
|  |  |  |  |  |
| Positive lymph nodes, N(%) | 20 (77%) | 10 (77%) | 10 (77%) | > 0.99 |
|  |  |  |  |  |
| Lymphovascular invasion (LVI), N(%) |  |  |  | 0.23 |
| No | 10 (45%) | 3 (30%) | 7 (58%) |  |
| Yes | 12 (55%) | 7 (70%) | 5 (42%) |  |
| Missing | 4 (15%) | 3 (23%) | 1 (8%) |  |
|  |  |  |  |  |
| Perineural spread/invasion (PNS), N (%) | 22 (85%) | 11 (85%) | 11 (85%) | > 0.99 |
|  |  |  |  |  |
| Pre-trial radiation therapy, N (%) |  |  |  | 0.48 |
| None | 8 (31%) | 5 (38%) | 3 (23%) |  |
| Neo-adjuvant only | 7 (27%) | 2 (15%) | 5 (38%) |  |
| Adjuvant only | 11 (42%) | 6 (46%) | 5 (38%) |  |
|  |  |  |  |  |
| Pre-trial systemic therapy, N(%) |  |  |  | 0.43 |
| Neo-adjuvant or adjuvant only | 15 (58%) | 9 (69%) | 6 (46%) |  |
| Both neo-adjuvant and adjuvant | 11 (42%) | 4 (31%) | 7 (54%) |  |
|  |  |  |  |  |
| Neo-adjuvant systemic therapy |  |  |  | 0.43 |
| None | 15 (58%) | 9 (69%) | 6 (46%) |  |
| 5-FU based combination | 11 (42%) | 4 (31%) | 7 (54%) |  |
|  |  |  |  |  |
| Adjuvant systemic therapy, N (%) |  |  |  | 0.041 |
| 5-FU based combination | 10 (38%) | 2 (15%) | 8 (62%) |  |
| Other | 16 (62%) | 11 (85%) | 5 (38%) |  |
|  |  |  |  |  |
| Progressive disease type, N (%) |  |  |  | 0.14 |
| Local only | 13 (50%) | 7 (54%) | 6 (46%) |  |
| Distant only | 6 (23%) | 1 (8%) | 5 (38%) |  |
| Local and distant | 4 (15%) | 2 (15%) | 2 (15%) |  |
| Clinical only | 3 (12%) | 3 (23%) | 0 (0%) |  |
|  |  |  |  |  |
| CA 19-9 at progression |  |  |  |  |
| Median (Q1, Q3) | 159.6 (38.1, 530.1) | 198.2 (137.1, 540.4) | 92.5 (30.0, 500.0) | 0.34 |
| >= 100, N(%) | 15 (62%) | 9 (82%) | 6 (46%) | 0.10 |
| Missing | 2 (8%) | 2 (15%) | 0 (0%) |  |
|  |  |  |  |  |
| Time from randomization to end of maintenance |  |  |  |  |
| Median (Q1, Q3) | 5.4 (2.8, 11.2) | 5.3 (2.8, 10.2) | 5.9 (2.6, 11.5) | 0.9 |
|  |  |  |  |  |
| Metastatic systemic therapy, N(%) |  |  |  | 0.017 |
| 5-FU based combination | 13 (50%) | 10 (77%) | 3 (23%) |  |
| Other | 13 (50%) | 3 (23%) | 10 (77%) |  |

**Table S3:** Best Response to Systemic Therapy in Advanced Disease Setting

| **Outcome** | **Total**  **(N = 26)** | **Observation**  **(N = 13)** | **CC-486**  **(N=13)** |
| --- | --- | --- | --- |
| **Best response**  Complete response  Partial response  Stable disease  Progressive disease  Biochemical response  Missing* | 0  1 (4%)  17 (65%)  5 (19%)  2 (8%)  1 (4%) | 0  0  9 (64%)  2 (14%)  1 7%)  1 (7%) | 0  1 (7%)  8 (57%)  3 (21%)  0  1 (7%) |

* 2 individuals were not evaluable for response: withdrew from follow-up (CC-486) and missing (OBS).

**Table S4:** Association between metastatic progression free survival and characteristics of interest among participants receiving chemotherapy in the advanced disease setting

| **Characteristic** | N | Events | Median | (95% CI) | 6-month MPFS | (95% CI) | HR | (95% CI) | P-value |
| --- | --- | --- | --- | --- | --- | --- | --- | --- | --- |
| Overall | 25 | 23 | 6.1 | (4.9-9.2) | 52% | (31%-70%) |  |  |  |
|  |  |  |  |  |  |  |  |  |  |
| Assigned treatment |  |  |  |  |  |  |  |  |  |
| Observation | 13 | 11 | 9.1 | (4.9-19.1) | 69% | (37%-88%) | 1 |  | 0.049 |
| CC-486 | 12 | 12 | 5.0 | (3.4-8.8) | 33% | (10%-59%) | 2.49 | (1.00-6.18) |  |
|  |  |  |  |  |  |  |  |  |  |
| Eligibility CA19-9 |  |  |  |  |  |  |  |  |  |
| No | 14 | 13 | 7.1 | (3.4-11.8) | 57% | (28%-78%) | 1 |  | 0.69 |
| Yes | 11 | 10 | 6.0 | (3.8-9.5) | 45% | (16%-71%) | 1.19 | (0.50-2.83) |  |
|  |  |  |  |  |  |  |  |  |  |
| Eligibility R1 |  |  |  |  |  |  |  |  |  |
| No | 20 | 18 | 7.1 | (4.2-9.5) | 60% | (35%-78%) | 1 |  | 0.11 |
| Yes | 5 | 5 | 5.0 | (2.0-Inf) | 20% | (0%-59%) | 2.29 | (0.81-6.43) |  |
|  |  |  |  |  |  |  |  |  |  |
| Eligibility node positive |  |  |  |  |  |  |  |  |  |
| No | 6 | 6 | 4.9 | (2.5-Inf) | 33% | (4%-68%) | 1 |  | 0.11 |
| Yes | 19 | 17 | 7.8 | (4.2-11.8) | 58% | (33%-77%) | 0.45 | (0.16-1.21) |  |
|  |  |  |  |  |  |  |  |  |  |
| Age |  |  |  |  |  |  |  |  |  |
| Less than 60 | 6 | 6 | 4.9 | (2.0-Inf) | 17% | (0%-52%) | 1 |  | 0.65 |
| 60 or older | 19 | 17 | 7.8 | (4.2-9.7) | 63% | (37%-81%) | 0.79 | (0.28-2.20) |  |
|  |  |  |  |  |  |  |  |  |  |
| Gender |  |  |  |  |  |  |  |  |  |
| Male | 14 | 12 | 8.8 | (5.0-11.8) | 71% | (40%-89%) | 1 |  | 0.14 |
| Female | 11 | 11 | 4.9 | (2.5-6.5) | 27% | (6%-54%) | 1.89 | (0.80-4.47) |  |
|  |  |  |  |  |  |  |  |  |  |
| ECOG at randomization |  |  |  |  |  |  |  |  |  |
| 0 | 19 | 17 | 6.4 | (3.9-9.7) | 53% | (28%-72%) | 1 |  | 0.39 |
| 1 | 6 | 6 | 5.5 | (2.5-Inf) | 50% | (11%-81%) | 1.53 | (0.58-4.01) |  |
|  |  |  |  |  |  |  |  |  |  |
| CA19-9 at randomization |  |  |  |  |  |  |  |  |  |
| < 100 | 22 | 20 | 6.3 | (4.2-9.7) | 55% | (32%-73%) | 1 |  | 0.31 |
| >= 100 | 3 | 3 | 4.9 | (3.8-Inf) | 33% | (0%-78%) | 1.92 | (0.54-6.74) |  |
|  |  |  |  |  |  |  |  |  |  |
| Surgical Resection Margin Status, N(%) |  |  |  |  |  |  |  |  |  |
| R0 | 20 | 18 | 7.1 | (4.2-9.5) | 60% | (35%-78%) | 1 |  | 0.11 |
| R1 | 5 | 5 | 5.0 | (2.0-Inf) | 20% | (0%-59%) | 2.29 | (0.81-6.43) |  |
|  |  |  |  |  |  |  |  |  |  |
| Histologic grade |  |  |  |  |  |  |  |  |  |
| Well/moderately differentiated | 22 | 20 | 6.0 | (3.9-9.7) | 50% | (28%-69%) | 1 |  | 0.55 |
| Poorly differentiated | 3 | 3 | 6.4 | (4.9-Inf) | 67% | (5%-95%) | 1.47 | (0.41-5.19) |  |
|  |  |  |  |  |  |  |  |  |  |
| T stage |  |  |  |  |  |  |  |  |  |
| T1/T2 | 16 | 15 | 6.3 | (3.8-9.7) | 56% | (29%-77%) | 1 |  | 0.55 |
| T3/T4 (involves celiac axis, SM or common hepatic artery) | 9 | 8 | 6.0 | (2.0-9.2) | 44% | (13%-72%) | 1.32 | (0.53-3.29) |  |
|  |  |  |  |  |  |  |  |  |  |
| Lymphovascular invasion (LVI) |  |  |  |  |  |  |  |  |  |
| No | 10 | 10 | 5.0 | (2.5-8.8) | 40% | (12%-68%) | 1 |  | 0.43 |
| Yes | 11 | 10 | 6.4 | (3.4-13.6) | 55% | (22%-78%) | 0.69 | (0.28-1.72) |  |
|  |  |  |  |  |  |  |  |  |  |
| Perineural spread/invasion (PNS) |  |  |  |  |  |  |  |  |  |
| No | 4 | 4 | 5.5 | (4.9-Inf) | 50% | (5%-85%) | 1 |  | 0.52 |
| Yes | 21 | 19 | 6.4 | (3.9-9.5) | 52% | (29%-71%) | 0.7 | (0.22-2.12) |  |
|  |  |  |  |  |  |  |  |  |  |
| Pre-trial radiation therapy, N(%) |  |  |  |  |  |  |  |  |  |
| None | 8 | 6 | 6.4 | (2.5-Inf) | 50% | (15%-78%) | 1 |  |  |
| Neo-adjuvant only | 7 | 7 | 6.1 | (3.8-9.7) | 57% | (17%-84%) | 0.99 | (0.32-3.02) | 0.99 |
| Adjuvant only | 10 | 10 | 6.2 | (2.0-11.8) | 50% | (18%-76%) | 0.94 | (0.32-2.72) | 0.91 |
|  |  |  |  |  |  |  |  |  |  |
| Pre-trial systemic therapy |  |  |  |  |  |  |  |  |  |
| Neo-adjuvant or adjuvant only | 14 | 12 | 7.8 | (2.5-11.8) | 57% | (28%-78%) | 1 |  | 0.36 |
| Both neo-adjuvant and adjuvant | 11 | 11 | 5.0 | (4.2-8.8) | 45% | (16%-71%) | 1.49 | (0.63-3.50) |  |
|  |  |  |  |  |  |  |  |  |  |
| Neo-adjuvant systemic therapy |  |  |  |  |  |  |  |  |  |
| None | 14 | 12 | 7.8 | (2.5-11.8) | 57% | (28%-78%) | 1 |  | 0.36 |
| 5-FU based combination | 11 | 11 | 5.0 | (4.2-8.8) | 45% | (16%-71%) | 1.49 | (0.63-3.50) |  |
|  |  |  |  |  |  |  |  |  |  |
| Adjuvant systemic therapy |  |  |  |  |  |  |  |  |  |
| 5-FU based combination | 10 | 10 | 7.1 | (3.4-9.7) | 60% | (25%-83%) | 1 |  | 0.76 |
| Other | 15 | 13 | 5.0 | (2.5-9.5) | 47% | (21%-69%) | 0.87 | (0.36-2.07) |  |
|  |  |  |  |  |  |  |  |  |  |
| CA 19-9 at progression |  |  |  |  |  |  |  |  |  |
| < 100 | 8 | 8 | 8.3 | (2.0-19.1) | 62% | (22%-87%) | 1 |  | 0.39 |
| >= 100 | 15 | 13 | 5.0 | (3.8-9.5) | 40% | (16%-63%) | 1.54 | (0.57-4.15) |  |
|  |  |  |  |  |  |  |  |  |  |
| Metastatic systemic therapy |  |  |  |  |  |  |  |  |  |
| 5-FU based combination | 12 | 11 | 7.6 | (2.0-13.6) | 58% | (27%-81%) | 1 |  | 0.34 |
| Other | 13 | 12 | 6.0 | (3.8-8.8) | 46% | (19%-70%) | 1.54 | (0.62-3.80) |  |
